# Photoresponsive Multirole Nanoweapon Camouflaged by Hybrid Cell Membrane Vesicles for Efficient Antibacterial Therapy of *Pseudomonas aeruginosa*‐Infected Pneumonia and Wound

**DOI:** 10.1002/advs.202403101

**Published:** 2024-07-15

**Authors:** Hening Liu, Lu Tang, Yue Yin, Yuqi Cao, Cong Fu, Jingwen Feng, Yan Shen, Wei Wang

**Affiliations:** ^1^ State Key Laboratory of Natural Medicines Department of Pharmaceutics School of Pharmacy China Pharmaceutical University Nanjing 211198 P. R. China; ^2^ NMPA Key Laboratory for Research and Evaluation of Cosmetics China Pharmaceutical University Nanjing 211198 P. R. China

**Keywords:** biomimetic nanomedicine, *P. aeruginosa*, pneumonia, synergistic antibacterial therapy, wound infection

## Abstract

Exploring effective antibacterial approaches for targeted treatment of pathogenic bacterial infections with reduced drug resistance is of great significance. Combinational treatment modality that leverages different therapeutic components can improve the overall effectiveness and minimize adverse effects, thus displaying considerable potential against bacterial infections. Herein, red blood cell membrane fuses with macrophage membrane to develop hybrid cell membrane shell, which further camouflages around drug‐loaded liposome to fabricate biomimetic liposome (AB@LRM) for precise antibacterial therapy. Specifically, photoactive agent black phosphorus quantum dots (BPQDs) and classical antibiotics amikacin (AM) are loaded in AB@LRM to accurately target the inflammatory sites through the guidance of macrophage membrane and long residence capability of red blood cell membrane, eventually exerting efficacious antibacterial activities. Besides, due to the excellent photothermal and photodynamic properties, BPQDs act as an efficient antibacterial agent when exposed to near‐infrared laser irradiation, dramatically increasing the sensitivity of bacteria to antibiotics. Consequently, the synergistic sterilizing effect produced by AB@LRM further restricts bacterial resistance. Upon laser irradiation, AB@LRM shows superior anti‐inflammatory and antibacterial properties in models of *P. aeruginosa*‐infected pneumonia and wounds. Hence, this light‐activatable antibacterial nanoplatform with good biocompatibility presents great potential to advance the clinical development in the treatment of bacterial infections.

## Graphical abstract

This work develops a multifunctional and biomimetic nanoplatform with excellent inflammation targetability and long circulation ability for synergistic antibacterial therapies. Owing to the modification of hybrid cell membrane on drug‐loaded liposome, the designed biomimetic nanoparticles can effectively mediate photothermal therapy, photodynamic therapy and chemotherapy, which shows desirable therapeutic efficacy against *Pseudomonas aeruginosa*‐infected pneumonia and wound.

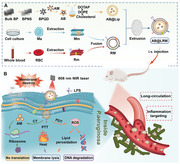

## 1 Introduction

Bacterial infections have emerged as one of the primary causes of mortality, becoming a prominent risk to human health worldwide.^[^ ^1^ ^]^ As a critical pathogen, *Pseudomonas aeruginosa* (*P. aeruginosa*), a gram‐negative bacterium, induces at least 10% of acquired infections globally.^[^ ^2^ ^]^ Over the last few decades, traditional antibiotic therapy has proved to be the most useful strategy for combating bacterial infection.^[^ ^3^ ^]^ However, antibiotic therapy shows the potential risk of toxicity and promotes the development of bacterial resistance.^[^ ^4^ ^]^ Besides, heritable drug‐resistant bacteria appear as a result of antibiotic misuse and show the terrible potential to result in a situation where there is no medication available in the future.^[^ ^5^ ^]^ Consequently, it is extremely urgent to create safe and precise antibacterial therapeutics for the treatment of *P. aeruginosa*‐infected diseases.

Currently, photothermal therapy (PTT) has received widespread attention for antibacterial application owing to its advantages of spatiotemporal controllability, minimal invasiveness, effective sterilization, and avoidance of bacterial resistance.^[^ ^6^ ^]^ Various nanomaterials including noble metal nanoparticles (NPs), metal oxides, carbon‐based nanostructures and polymers were reported to induce effective PTT.^[^ ^7^ ^]^ These excellent pioneering investigations revealed the brilliant prospects of phototherapy‐based multifunctional nanoplatforms in treating diverse diseases.^[^ ^8^ ^]^ However, complete eradication of bacteria using PTT alone usually requires a higher local temperature over 70 °C, which can cause inflammation and thermal damage to surrounding normal tissues.^[^ ^9^ ^]^ Therefore, combining PTT with other strategies shows the advantage by enhancing the efficacy of antibacterial treatment while minimizing hyperthermia‐related adverse effects. Black phosphorus quantum dots (BPQDs) stand out among these photoactive nanomaterials due to their broad optical absorption, high photothermal conversion efficiency, and favorable biocompatibility.^[^ ^10^ ^]^ It is worth noting that BPQDs are fully biodegradable within the body, endowing them with huge potential to be used as reliable PTT agents for multiple therapeutic purposes. On the other hand, BPQDs serve as efficient photosensitizers to transfer endogenous dioxygen into the cytotoxic reactive oxygen species (ROS) under NIR irradiation, which can be utilized as excellent candidate in photodynamic therapy (PDT).^[^ ^11^ ^]^ Although BPQDs individually are capable of performing PDT and PTT upon 808 nm laser irradiation to achieve efficient sterilization in vitro, they are extremely sensitive to oxygen and exhibit poor photostability in physiological environment, which significantly limits their effectiveness in vivo.^[^ ^12^ ^]^ To overcome these challenges, exploring feasible and effective nanostrategies to protect BPQDs from degradation during in vivo therapy and endow them with targeting ability towards desired site of action is of great significance.

Additionally, synthetic nanomaterials are frequently eliminated by the immune system swiftly, which dramatically reduce their drug administration efficiency.^[^ ^13^ ^]^ Importantly, enabling nanocarriers to evade the immune system and extending their circulation time to deliver antibiotics play key roles in reducing bacterial resistance.^[^ ^14^ ^]^ The combination of synthetic nanocarriers with natural cell membranes has been used in many disease models such as cancer and inflammation treatment.^[^ ^15^ ^]^ Particularly, these biomimetic NPs display good biocompatibility, immune escape ability and superior targeting capability inherited from their parent cells.^[^ ^16^ ^]^ In this area, red blood cell (RBC)‐derived nanocarriers have exhibited enormous potential in various pre‐clinical researches because of their abundant source and long life span in blood circle.^[^ ^17^ ^]^ RBC membrane (Rm) expressing many immunomodulatory markers such as CD47 has been employed to decorate NPs to prolong their plasma circulation in the body.^[^ ^18^ ^]^ As the primary line of defense to resist bacterial infections, macrophage (Ma) can recognize and engulf microbial organisms through toll‐like receptors. TLR2 and TLR4 receptors on Ma membrane (Mm) can bind to bacteria specifically.^[^ ^19^ ^]^ On this basis, fabricating hybrid cell membrane (RM) coating by fusing Rm with Mm shows great promise in targetedly delivering NPs to bacterial infection regions in the body, which not only enables the designed NPs to escape from immune clearance but also promotes the accumulation of drug‐loaded NPs in inflammation sites.^[^ ^20^ ^]^ Meanwhile, compared to single cell membrane coating, hybrid cell membrane coating combines the unique features of both single membrane components and inherit the functions of each cell type to accomplish multiple functions, which is beneficial to improve the overall therapeutic outcome.

**Scheme 1:**
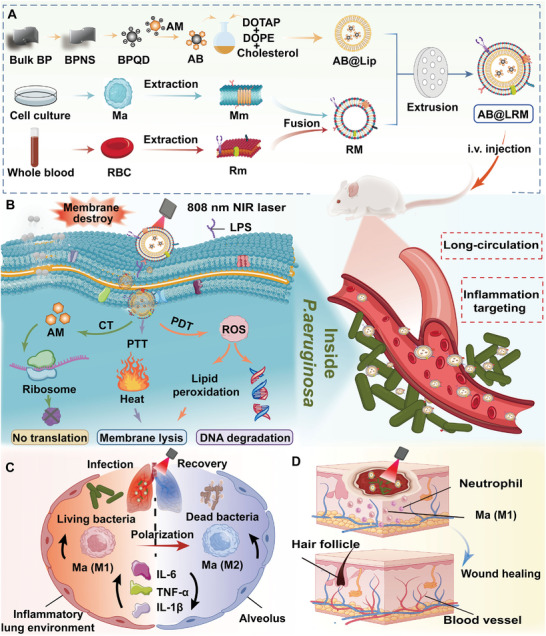
Schematic illustration of the construction of AB@LRM NPs and their antibacterial mechanisms. A) The preparation process of AB@LRM NPs. B) The synergistic therapeutic mechanisms against bacterial infections. Applications of AB@LRM (+) in the treatment of C) bacterial pneumonia and D) bacteria‐infected wound healing caused by *P. aeruginosa*.

For this reason, a near‐infrared (NIR) light‐responsive nanomedicine co‐loading classical antibiotics amikacin (AM) and photoactive agent BPQDs was designed in this work for precise antibacterial therapy (**Scheme** **1**). First of all, positively charged AM was absorbed with negatively charged BPQDs through an electrostatic reaction and liposomes served as the nanocarriers to deliver BPQDs and AM (AB@Lip). Then, the membranes of RBC and Ma were separately extracted and fused to form hybrid membranes, which further decorated around AB@Lip to obtain AB@LRM NPs. After intravenous (i.v.) injection, AB@LRM NPs precisely recognized bacteria and effectively accumulated in infection sites due to the targeting function of Mm and the long circulation performance of Rm. Under NIR irradiation, BPQDs produced ROS and heat, which enhanced the thermal sensitivity of the targeted bacteria and damaged their structure.^[^ ^21^ ^]^ More importantly, the membrane‐disruption effects of BPQDs facilitated the penetration of AM into bacteria to achieve sterilization effect with high efficiency owing to the clever combination of phototherapy with chemotherapy. Bacterial pneumonia model infected by *P. aeruginosa* was established to verify the therapeutic efficacy of AB@LRM plus laser irradiation (AB@LRM (+)), which revealed that adopting this strategy could significantly minimize the residual bacteria in lung tissue, relieve pulmonary edema, and ameliorate the inflammatory environment in the lung, exhibiting potent antibacterial effectiveness to rescue mice suffered from pulmonary infections and prolong their survival time. In another in vivo model based on wound infection, AB@LRM (+) efficaciously accelerated wound healing procedure, killed the bacteria in wound sites, and alleviated local inflammation, demonstrating its outstanding therapeutic efficacy against *P. aeruginosa*‐caused infections. Overall, this work offers a promising strategy by developing safe and efficient treatment methods based on nanomedicine against lethal bacterial infections, which might facilitate the clinical advancement in this field.

## 2 Results and Discussion

### 2.1 Construction and Characterization of AB@LRM NPs

**Figure 1:**
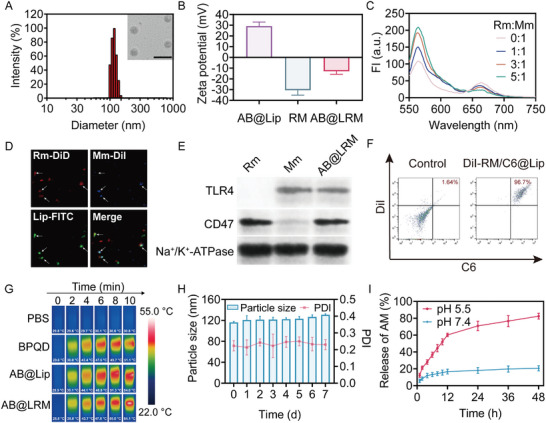
Characterizations of AB@LRM. A) TEM image and size distribution histogram of AB@LRM) (scale bar = 200 nm). B) Zeta potentials of AB@Lip, RM, and AB@LRM (*n* = 3). C) Study of membrane fusion by FRET experiment. D) Confocal laser scanning microscopy (CLSM) images of the co‐localization of different components in AB@LRM. E) Western blot analysis of key proteins on Rm, Mm and AB@LRM NPs. F) Determination of the coating efficiency of RM by FCM analysis. G) Infrared thermal imaging of AB@LRM at different time points under laser irradiation (808 nm, 1.5 W cm^−2^), BPQD concentration: 20 µg mL^−1^. H) Stability of AB@LRM over time in PBS containing 10% FBS (*n* = 3). I) Cumulative release rates of AM from AB@LRM at pH 7.4 and pH 5.5 (*n* = 3).

The construction process of AB@LRM was illustrated in Scheme 1. Briefly, BPQDs were synthesized by the liquid exfoliation method, and coencapsulated with AM in liposomes through thin‐film hydration method. Then, both Mm and Rm were extracted according to established protocols and coated on AB@Lip via co‐extrusion.^[^ ^22^ ^]^ A transmission electron microscope (TEM) was used to characterize the morphology of synthesized BPQDs and AB@LRM. BPQDs were monodispersed with an average diameter of around 20 nm, indicating their ultra‐small size (Figure S1, Supporting Information). Meanwhile, the X‐ray diffraction (XRD) was used to testify the crystal form of BPQDs. The diffraction peaks at 2*θ* = 16°, 34° and 52° corresponded well to the crystal surfaces included (020), (040), and (060), demonstrating that the prepared BPQDs had good crystallinity (Figure S2, Supporting Information).^[^ ^21^ ^]^ After fusion with RM vesicles, AB@Lip was camouflaged to obtain AB@LRM with spherical structure in uniform morphology. Additionally, AB@LRM showed a thin RM shell, which confirmed the coating of AB@Lip by RM vesicles (**Figure** **1**A). Furthermore, the particle size of AB@Lip determined using dynamic light scattering (DLS) was 97.0 ± 3.6 nm (Figure S3, Supporting Information). The particle size of AB@LRM was found to be 116.4 ± 2.9 nm, indicating the successful coating of RM vesicles around AB@Lip (Figure 1A). Meanwhile, the zeta potentials of AB@Lip and RM vesicles were 29.2 ± 3.8 mV and −30.6 ± 4.6 mV, respectively. However, the zeta potential of AB@LRM changed to −13.0 ± 2.7 mV after coating with RM vesicles, further verifying the efficient coating of RM vesicles surrounding AB@Lip (Figure 1B).

To verify the membrane fusion process, DiD and DiI, a dye pair with the Förster resonance energy transfer (FRET) property, were utilized to label Mm, and then fused with unlabeled Rm.^[^ ^22^ ^]^ By raising the weight ratio of Rm to Mm, fluorescence intensity at 565 nm was recovered, while the fluorescence at 663 nm gradually declined (Figure 1C). This result demonstrated that fusing two cell membranes weakened the FRET connection in the original Mm, demonstrating the successful membrane fusion process. Besides, fluorescent co‐localization experiment was performed to prove the fabrication of AB@LRM. A remarkable colocalization of red, blue and green fluorescent signals was viewed in AB@LRM NPs under CLSM, revealing that the fused RM membrane was coated on drug‐loaded liposome (Figure 1D). Ensuring the existence of RBC and Ma protein on AB@LRM is essential to guarantee its targeting and long circulation ability. According to SDS‐PAGE analysis, it was verified that AB@LRM retained typical proteins in both membranes (Figure S4, Supporting Information). Moreover, a western blot assay was carried out to confirm the existence of intact TLR4, a key protein of Ma, and CD47, a key protein of RBC, in AB@LRM (Figure 1E).^[^ ^23^ ^]^ In addition, the composition of AB@LRM was determined by flow cytometry (FCM) analysis. The fluorescence intensity of coumarin 6 (C6) and DiI in DiI‐RM/C6‐AB@Lip considerably enhanced in comparison to the control group, which was detected to be 96.7% in DiI‐RM/C6‐AB@Lip NPs, proving that AB@Lip was effectively clothed by RM vesicles (Figure 1F; Figure S5, Supporting Information). In summary, these results demonstrated that the hybrid membrane was successfully wrapped on the surface of AB@LRM to obtain biomimetic NPs.

The PTT performance is very important to achieve effective bacteria‐killing outcome, therefore, the PTT efficacy of different formulations was evaluated in vitro. To begin with, the UV–vis spectra of BPQDs and AB@LRM were scanned, which showed that AB@LRM possessed extensive absorption from UV to NIR regions, and this spectrum was consistent with that of BPQDs, indicating that the photothermal properties of BPQDs were not affected after encapsulation in biomimetic liposomes (Figure S6, Supporting Information). Subsequently, the photothermal effect and photothermal stability of AB@LRM upon 808 nm laser irradiation were evaluated by monitoring the solution temperature within 10 min. Compared with PBS, the temperature of AB@LRM increased as fast as BPQDs within 10 min (Figure S7, Supporting Information). Furthermore, the real‐time photothermal images of PBS, BPQDs, AB@Lip and AB@LRM were captured, which directly reflected the excellent PTT effect of AB@LRM that could climb to 54.1 °C (Figure 1G). In addition, the photothermal conversion of AB@LRM was found to be concentration‐ and power intensity‐dependent (Figures S8 and S9, Supporting Information). Statistically, the photothermal conversion efficiency of AB@LRM was determined to be 33.8% (Figure S10, Supporting Information). The photothermal stability is extremely important because AB@LRM will be exposed to physiological circumstances prior to PTT. Thus, we investigated whether the stability of BPQDs would be improved after encapsulation in biomimetic liposomes compared to free BPQDs. As revealed, the temperature of BPQDs increased by approximately 26.9 °C after irradiation for 10 min on day 0, however, the temperature only rose by 14.9 °C after 5 d. On the other hand, AB@LRM was more photothermally stable, and the temperature increased by 24.8 °C even after 5 d (Figure S11, Supporting Information). This finding suggested that encapsulating BPQDs through nanocarriers could successfully postpone their degradation to maintain their photothermal characteristics. Furthermore, the steady temperature increase after five cyclical irradiations also revealed that AB@LRM had strong photothermal stability (Figure S12, Supporting Information). Taken together, AB@LRM was demonstrated to produce effective PTT performance because of its outstanding photostability and great photothermal conversion efficiency.

To examine the stability of AB@LRM in vitro, AB@LRM was dispersed in PBS or PBS containing 10% fetal bovine serum (FBS) and stored for 7 d. The hydrodynamic diameter and polydispersity index (PDI) were measured once a day, which showed that these parameters did not experience severe fluctuations over 7 d, indicating that AB@LRM could remain stable during in vivo transportation (Figure 1H; Figure S13, Supporting Information). To investigate the release characteristics of AM from AB@LRM, pH 5.5 and pH 7.4 were utilized to imitate an acidic infection microenvironment and a neutral blood circulation environment, respectively.^[^ ^24^ ^]^ As the release curve indicated, AB@LRM gradually released about 80% of AM in 48 h at pH 5.5. In contrast, only about 20% of AM was released from AB@LRM at pH 7.4, suggesting that it was advantageous for AM to be released from AB@LRM in an acidic infected location compared to neutral physiological condition (Figure 1I). Moreover, the encapsulation efficiency (EE) and drug loading efficiency (DLE) of AM and BPQDs in AB@LRM NPs were detected to be 37.51 ± 2.67% (EE) and 4.64 ± 0.33% (DLE), and 50.24 ± 3.61% (EE) and 5.18 ± 0.37% (DLE), respectively, verifying the effective co‐encapsulation of both therapeutic agents in designed NPs.

### 2.2 Antibacterial Efficacy In Vitro

**Figure 2:**
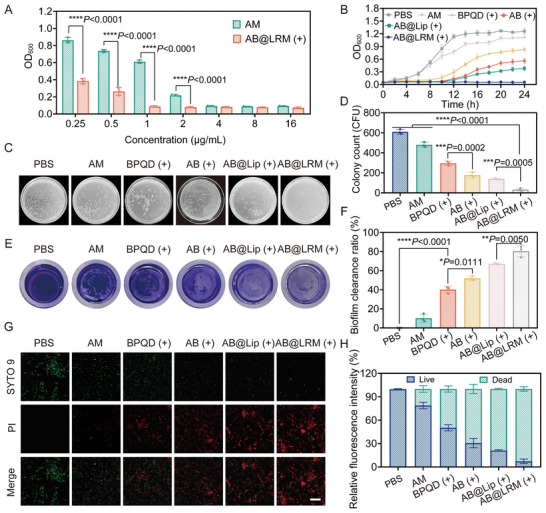
Antibacterial properties of AB@LRM (+) in vitro. A) The antibacterial effects of AM, AB@LRM (+) against *P. aeruginosa* at a series of AM concentrations (*n* = 3). B) Growth curves of *P. aeruginosa* after different treatments at the same AM concentration of 1 µg mL^−1^ (*n* = 3). C,D) Representative photographs and their quantitative analysis of bacterial colonies formed on agar plates in different treatment groups (*n* = 3). E,F) Biofilm biomass of *P. aeruginosa* identified by crystal violet staining after various treatments and the quantitative evaluation of biofilm clearance ratio (*n* = 3). G,H) Live/dead staining images of *P. aeruginosa* after various treatments (scale bar = 20 µm) and the matching quantitative analysis of live and dead bacteria (*n* = 3). *p < 0.05, **p < 0.01, ***p < 0.001, ****p < 0.0001.

Due to its consistently high morbidity and mortality rates, *P. aeruginosa* has emerged as one of the most widespread bacteria and continues to pose a serious threat to public health.^[^ ^25^ ^]^ Prompted by the above‐mentioned properties, the antibiosis of NIR‐irradiated AB@LRM against *P. aeruginosa* was investigated in vitro. To begin with, the micro‐broth dilution method was used to assess the minimum inhibitory concentration (MIC) of free AM and AB@LRM (+) against *P. aeruginosa*.^[^ ^26^ ^]^ The MIC of free AM was 4 µg mL^−1^. By contrast, the MIC of AM in AB@LRM (+) was only 1 µg mL^−1^, which was threefold lower than that of free AM (**Figure** **2**A). This result demonstrated that the combination therapy strategy significantly reduced the dosage of antibiotics, thereby reducing the possibility of bacterial resistance. The synergistic antibacterial activity of different treatment groups on *P. aeruginosa* was further examined through agar plate assay at the same AM concentration of 1 µg mL^−1^ (Figure 2C,D; Figure S14, Supporting Information). The PBS group showed an abundance of colonies on Luria‐Bertani (LB) agar. Compared to the PBS group, the relative antibacterial ratio in AM group was 21.0% and the colony count decreased slightly. By contrast, BPQDs, AB and AB@Lip inhibited *P. aeruginosa* growth to some extent when exposed to NIR radiation, and the relative antibacterial ratio was 51.3%, 70.9%, and 76.9%, respectively. Surprisingly, only 5.5% of bacteria survived in the AB@LRM group, which was probably due to the ability of Mm to adhere to bacterial surfaces through the identification of bacterial receptors, eventually generating effective PTT and PDT to cause powerful sterilizing efficacy. Moreover, bacteria became more sensitive to antibiotics due to the photothermal effect of BPQDs, which further improved the overall antibacterial activity. To assay the long‐term inhibitory effect, the growth curves of *P. aeruginosa* were recorded by monitoring the optical density of bacteria suspension at 600 nm for 24 h (Figure 2B). Similar to PBS, AM had no discernible influence on bacterial growth at 1 µg mL^−1^. However, BPQD (+) (20 µg mL^−1^) significantly delayed the bacterial growth in the first 10 h due to the photothermal effect and similar phenomena were observed in AB (+) and AB@Lip (+) groups owing to the combined effect of antibiotics and photothermal performance. Noticeably, bacterial growth was completely inhibited by AB@LRM (+), indicating its high antibacterial efficacy and long‐term bacteriostatic ability.

Persistent bacterial infection is intimately relevant to the development of the biofilm, which may lead to drug resistance and protect bacteria from the host immune system. Thus, destroying bacterial biofilms is more challenging than eliminating planktonic bacteria.^[^ ^27^ ^]^ The crystal violet staining method was employed to quantify the amount of biofilm clearance after AB@LRM (+) treatment (Figure 2E,F; Figure S15, Supporting Information). Compared to the PBS group, AM presented negligible ability to eliminate the biofilm, which was due to the fact that free AM could not easily penetrate the biofilm to employ its antibacterial properties. Conversely, 40.1% of the biofilm was removed in BPQD (+) group, reflecting a moderate effect produced by photothermal ablation on biofilm removal. Meanwhile, the biofilm clearance rate after AB (+) treatment was 51.9%, which was significantly higher than free BPQD (+) treatment. Apparently, the coexistence of AB@LRM and NIR irradiation eliminated biofilm biomass up to 80.3%, illustrating the most severe biofilm damage due to the combinational effects resulted from photothermal ablation and improved penetration behavior to promote AM release. Based on the above investigations, AB@LRM (+) was demonstrated to own an outstanding effect on the clearance of biofilm.

Subsequently, the live/dead bacterial staining method further visually verified the bactericidal effect of our designed nanoplatform. Live *P. aeruginosa* was labeled with SYTO 9 to display green fluorescence, while dead *P. aeruginosa* was stained by propidium iodide (PI) to show red fluorescence (Figure 2G).^[^ ^28^ ^]^ CLSM images showed that almost all the bacteria were stained with strong green fluorescence after treatment with PBS. However, treatment using AM, AB (+), and AB@Lip (+) simultaneously emitted both green and red fluorescence, indicating mild bactericidal efficacy. Of note, bacteria in AB@LRM (+) group displayed extremely weak green fluorescence and significant red fluorescence. The quantitative measurement of fluorescence intensity using Image J software directly showed that AB@LRM (+) had the highest antibacterial performance compared to other groups (Figure 2H).

### 2.3 Exploration of the Antibacterial Mechanism

**Figure 3:**
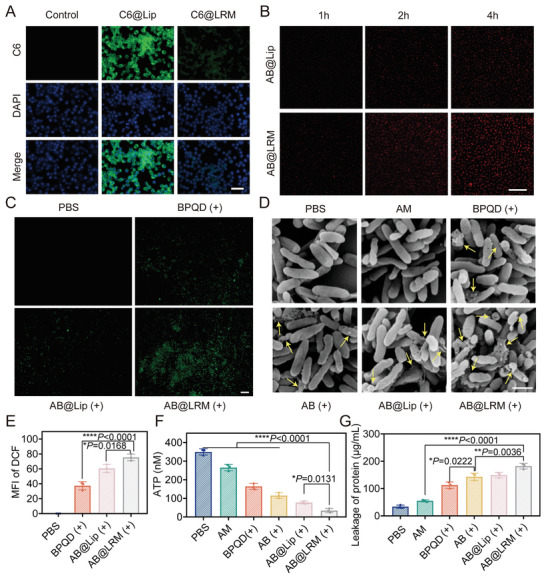
Antibacterial properties of AB@LRM (+) in vitro. A) Cellular uptake of AB@Lip NPs and AB@LRM NPs by RAW264.7 cells, respectively (scale bar = 50 µm). B) CLSM images of bacteria after incubation with AB@Lip or AB@LRM NPs at 37 °C for 1, 2, and 4 h, respectively (scale bar = 20 µm). C,E) Fluorescence images of intracellular ROS production in *P. aeruginosa* detected by DCFH‐DA probe after various treatments (scale bar = 50 µm) and their quantitative analysis (*n* = 3). D) SEM images of bacteria after various treatments (scale bar = 1 µm). F,G) ATP level and protein leakage in bacteria suspensions after different treatments (*n* = 3). *p < 0.05, **p < 0.01, ****p < 0.0001.

To investigate the possible antibacterial mechanism of AB@LRM NPs, a series of experiments were performed accordingly. First, the immune escape ability of nanosystems is crucial for prolonging the circulation time in vivo to achieve targeted drug delivery.^[^ ^29^ ^]^ To illustrate the immune escape capability of AB@LRM in vitro, its engulfment efficiency by RAW264.7 cells was assessed using C6‐labeled LRM and observed under an inverted fluorescence microscope (IFM).^[^ ^30^ ^]^ Compared to AB@Lip group, a significantly reduced green fluorescence in RAW264.7 cells was observed in AB@LRM group, and its quantification analysis results also corresponded to the IFM observations (**Figure** **3**A; Figure S16, Supporting Information). Therefore, AB@LRM well inherited the immunological characteristics of hybrid membranes, which could decrease its immunogenicity and effectively inhibit the phagocytosis by phagocytotic cells.

The surface of Mm contained TLR4 receptors, which allowed AB@LRM to specifically target *P. aeruginosa*. The interactions of DiI‐labeled AB@LRM with bacteria at different time were investigated by CLSM (Figure 3B). It was interesting to find that after the same incubation time, the fluorescence in the bacteria treated with AB@LRM NPs was noticeably stronger than that in AB@Lip group. The strong fluorescence revealed the accumulation and adhesion ability of AB@LRM NPs in the bacterial regions, which laid the groundwork for the potent antibacterial activity. Meanwhile, BPQDs functioned as photosensitizers and produced large amounts of ROS species upon laser irradiation, leading to lipid peroxidation and membrane destruction. The electron spin resonance (ESR) experiment was performed to confirm the ROS species generated by BPQDs and AB@LRM NPs after laser irradiation, which showed that BPQDs and AB@LRM NPs clearly displayed the typical 1:1:1 triplet signal, confirming that singlet oxygen (^1^O_2_) was the predominant ROS type generated by BPQDs (Figure S17, Supporting Information). Then, 2′, 7′‐dichlorodihydrofluorescein (DCFH‐DA), which changes into 2′, 7′‐dichlorofluorescein (DCF) with bright green fluorescence by the oxidation of ROS, was utilized to measure the intracellular ROS level after different treatments.^[^ ^31^ ^]^ Green fluorescence was rarely detected following PBS treatment, indicating no ROS induction. Whereas in BPQDs or AB@Lip‐treated group, laser irradiation apparently increased ROS production. At the same time, bacteria treated with AB@LRM (+) produced higher levels of ROS, suggesting that AB@LRM successfully bonded to the bacterial surface and released high degree of BPQDs into bacteria (Figure 3C,E). More importantly, drug resistance would not be induced through this route.^[^ ^32^ ^]^ Subsequently, the bacterial morphology after different treatments was examined using a scanning electron microscope (SEM) (Figure 3D).^[^ ^33^ ^]^ Yellow arrows in the images represented the obvious membrane damage and content leakage. SEM images indicated that *P. aeruginosa* exhibited a uniform rod‐like shape with a smooth surface as displayed in control group. By contrast, after treatment with AM, the bacterial surface was slightly damaged and wrinkled. Under NIR laser irradiation, the bacteria treated with BPQDs showed obvious damage on their surface. Furthermore, most of *P. aeruginosa* contents leaked out and the damage was severer after treatment with AB@LRM (+), confirming the strong bacterial damaging effect of AB@LRM under NIR light irradiation. Since adenosine triphosphate (ATP) and protein are important intracellular components, their discharge is regarded as the typical sign of cell membrane deterioration.^[^ ^34^ ^]^ Therefore, the changes of ATP levels within bacteria were explored (Figure 3F). Upon NIR laser irradiation, the decrease of ATP levels in bacteria cultured with AB@LRM was the highest among all treatment groups, reaching up to 90.3%. Similarly, AB@LRM treatment showed a large amount of protein leakage after irradiation, causing a 4.4‐fold increase in protein leakage from bacteria compared with that in PBS group (Figure 3G). These findings demonstrated that laser‐assisted AB@LRM resulted in severe destruction of bacterial cell membranes. In addition, the internal leakage of bacteria provided strong evidence to verify its direct bacterial killing efficiency. In conclusion, AB@LRM (+) could kill *P. aeruginosa* by multiple synergistic mechanisms including great targeting ability towards *P. aeruginosa*, elevated ROS level and considerable damage to cell membrane integrity.

### 2.4 Antibacterial Property of AB@LRM (+) in the Infected Wound Model

**Figure 4:**
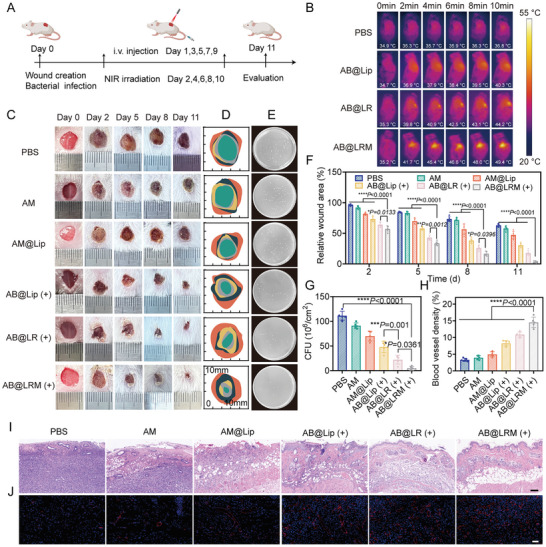
Antibacterial potential of AB@LRM (+) in the *P. aeruginosa*‐infected wound model in vivo. A) Schematic illustration of the establishment and assessment of bacterial infected full‐thickness cutaneous wound. B) Real‐time infrared thermal photos of mice following various treatments under NIR exposure. C) Representative photographs of infected wounds on days 0, 2, 5, 8, and 11. D) Monitoring of wound closure during 11 d treatments in each group. E,G) Representative photographs of bacterial colonies and quantitative measurement of CFU in *P. aeruginosa*‐infected wounds (*n* = 5). F) The relative wound area in different groups (*n* = 5). H,J) Representative immunofluorescence images of angiogenesis (CD31) (scale bar = 50 µm) and the quantifications of vessel density (*n* = 5). I). H&E staining of the infected wound tissues (scale bar = 100 µm). *p < 0.05, **p < 0.01, ***p < 0.001, ****p < 0.0001.

To further indicate the antibacterial property of AB@LRM, the in vivo model of *P. aeruginosa*‐infected wound was established.^[^ ^35^ ^]^ After 24 h, mice were randomly assigned to six groups and treated with different formulations by i.v. injections once every 2 days (**Figure** **4**A). For laser‐irradiated groups, the infection area received a NIR irradiation (1.5 W cm^−2^, 10 min) every other day. Meanwhile, the photothermal effect was evaluated using thermal imaging equipment (Figure 4B; Figure S18, Supporting Information). The local temperature of mice in AB@LRM group showed the fastest temperature increase to 49.4 °C after irradiation by NIR light for 10 min, which has been deemed high enough to destroy bacteria according to previous reports.^[^ ^36^ ^]^ Moreover, the temperature of AB@LRM group was considerably higher than that of the other groups, confirming the excellent PTT efficacy and the longest retention time of BPQDs at infection sites. The wound area was recorded daily throughout the entire treatment period. After different treatments for 11 d, the wound treated with AB@LRM (+) showed a noticeably faster healing effect than the other groups (Figure 4C,D). Additionally, the wound size was evaluated to assess the wound healing procedure. The wound area of the AB@LRM (+) group decreased from the initial wound to 3.4%, while the PBS group only decreased to 62.7%, demonstrating the potency of AB@LRM as a wound healing promoter (Figure 4F). Subsequently, to investigate whether the quick healing of wounds was resulted from the extensive eradication of bacteria, the wound tissues were collected and grounded for colony‐forming unit (CFU) counting on day 11 (Figure 4E,G). Compared with the other groups, CFU obviously decreased after treatment with AB@LRM (+) and the number of bacteria colonies decreased to 4.1%, proving that the local infections was successfully relieved by this multifunctional synergistic therapy based on AB@LRM (+). Furthermore, hematoxylin and eosin (H&E) staining was used for the histological evaluation of the wound section in order to assess the healing effect following different treatments (Figure 4I). H&E staining images showed that there were obvious inflammation reactions and abundant inflammatory cells in the wound of the PBS group. By contrast, infected areas treated with AB@LRM (+) demonstrated a significant decrease in inflammatory cells as well as complete re‐epithelialization of wound sites with typical skin morphological features of hair follicles and blood vessels, implying the effective control of inflammation. Furthermore, the degree of tissue regeneration and skin function recovery is reflected by neovascularization. Therefore, immunofluorescence was utilized to assess the neovascularization at the wound sites based on the expression of CD31, a biomarker of endothelial cells (Figure 4H,J).^[^ ^37^ ^]^ AB@LRM (+) group showed the best formation of the blood vessel and the blood vessel density in the wound site, which increased 3.4 times compared to the PBS group, implying an improved angiogenesis and tissue repair in the wounds. Overall, the in vivo studies discussed above verified that our designed AB@LRM combined with laser treatment could act as an effective weapon against *P. aeruginosa*‐caused wounds and displayed great potential to hasten wound healing.

### 2.5 Transcriptomic Analysis after AB@LRM (+) Treatment in Infected Wound Model

**Figure 5:**
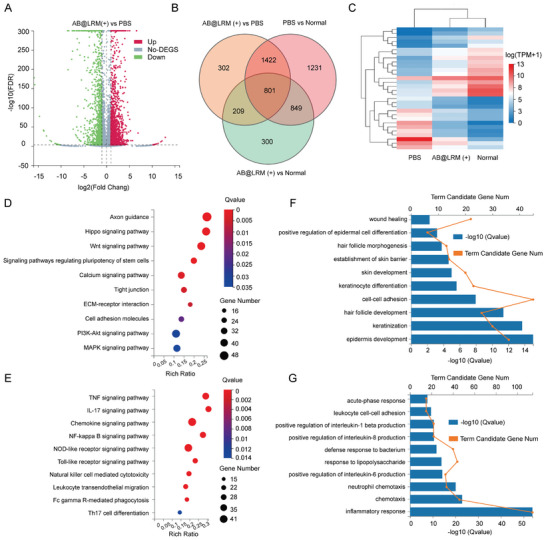
Transcriptomic analysis illustrating the therapeutic mechanism of AB@LRM (+) in relieving wound infection. A) Volcano plot showing DEGs in PBS and AB@LRM (+) groups. B) Venn diagrams illustrating the number of DEGs in AB@LRM (+) versus PBS, PBS versus normal, and AB@LRM (+) versus normal. C) Heatmap of hierarchical clustering of DEGs. D) Upregulated and E) downregulated DEGs enriched in the KEGG pathway. GO enrichment analysis of the gene functions of F) upregulated and G) downregulated DEGs.

We further investigated the detailed mechanisms of AB@LRM (+) in promoting wound healing in genetic level by RNA‐sequencing (RNA‐seq) analysis of mice skin after treatments. RNA‐seq assay revealed that among the 17610 genes, 1682 (10%) genes were upregulated, while 1052 (6%) genes were downregulated in AB@LRM (+) group compared with the PBS group (| Log2(fold change) | ≥ 1, *P* < 0.05) (**Figure** **5**A; Figure S19, Supporting Information). After intersecting the differentially expressed genes (DEGs) of AB@LRM group, PBS group and normal skin group (normal group), we found that there were 801 coregulated genes (Figure 5B). Subsequently, the expression level of DEGs from the AN@LRM (+), PBS and normal groups were presented by cluster heatmap (Figure 5C). The expression patterns of the AB@LRM (+) group and the normal group were comparable, while the gene expression levels of the PBS group were significantly different from the other two groups. According to this analysis, the wound condition of mice treated with AB@LRM (+) was significantly improved and gradually tended to normal.

Furthermore, the two most adopted analysis methods, Kyoto Encyclopedia of Genes and Genomes (KEGG) and Gene Ontology (GO) were utilized to identify the functions of DEGs respectively.^[^ ^38^ ^]^ First, the main pathways of DEGs between the PBS and AB@LRM (+) groups were evaluated using the KEGG database. As one of the pathways involved in wound healing, the Wnt signaling pathway is considered to have many functions, such as regulating the development of skin and its appendages, inducing the morphogenesis of skin appendages, regulating the periodic growth of hair follicles, and promoting wound angiogenesis and epithelial remodeling.^[^ ^39^ ^]^ According to KEGG pathway analysis, upregulated genes were shown to be involved in the Wnt signaling pathway, suggesting that AB@LRM (+) promoted wound healing compared with the PBS group (Figure 5D). At the same time, through KEGG enrichment analysis of downregulated genes, we also discovered that the DEGs were primarily enriched in pathways involved in inflammation, such as NF‐kappa B signaling pathway, IL‐17 signaling pathway, and TNF signaling pathway. Accordingly, the inflammation level in the wound was downregulated after treatment with AB@LRM (+) (Figure 5E). The GO database was employed to identify DEGs enriched in the major function.^[^ ^40^ ^]^ According to GO enrichment analysis, the upregulation of genes was mainly related to epidermis development, hair follicle development, skin development, establishment of skin barrier and wound healing (Figure 5F; Figure S20A, Supporting Information). Moreover, the downregulation of genes is mostly associated with positive regulation of interleukin‐1 beta production, positive regulation of interleukin‐8 production, positive regulation of interleukin‐6 production and inflammatory response, etc. (Figure 5G; Figure S20B, Supporting Information). Overall, these findings based on transcriptomic analysis illustrated the positive function of AB@LRM (+) on inflammation alleviation and wound healing regulation.

### 2.6 Biodistribution and In Vivo Therapeutic Effect against Bacterial Pneumonia

**Figure 6:**
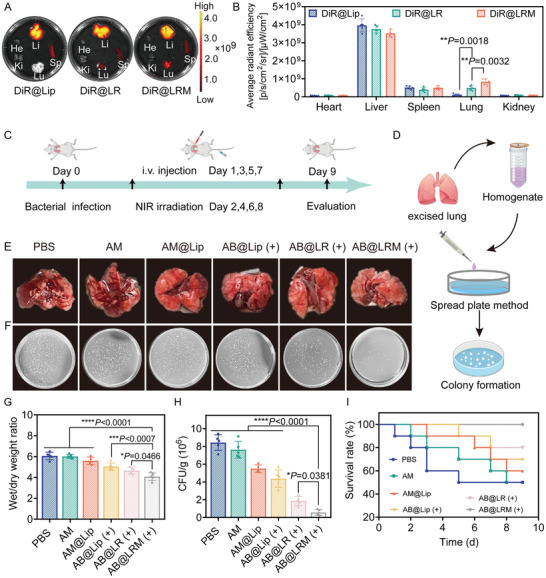
Antibacterial efficacy of AB@LRM (+) in bacterial pneumonia model infected by *P. aeruginosa*. A,B) Ex vivo fluorescence imaging and quantitative fluorescence intensity of the excised organs at 24 h post‐injection (*n* = 5). C) Schematic illustration of the experimental design. D) Diagram illustrating the procedures of bacteria calculation in lung tissues. E) Representative photographs of lungs harvested from mice in different treatment groups. F,H) Agar plate photographs and quantitative analysis of bacteria after different treatments in lungs (*n* = 5). G) Wet/dry weight ratio of lungs (*n* = 5). I) Survival curves of mice with bacterial pneumonia in various treatment groups (*n* = 10). *p < 0.05, **p < 0.01, ***p < 0.001, ****p < 0.0001.

Owing to the adherent ability of AB@LRM towards bacteria and its immune escape capability in vitro, we further testified its targeting ability in vivo. DiR‐labeled NPs were employed to track the biodistribution and plasma kinetics of different prepared NPs in vivo. Firstly, bacterial pneumonia mouse model was established and injected with different NPs intravenously. At predetermined time points, the blood of mice was collected to determine the fluorescence signal of DiR at different time. As the pharmacokinetic curves detected by IVIS indicated, hybrid LRM nanocarrier could effectively prolong the blood circulation of DiR, which showed obvious fluorescence signal at 24 h compared to DiR@Lip group (Figure S21, Supporting Information). Meanwhile, the targeting potential of LRM nanocarrier towards bacteria infected lung was investigated (**Figure** **6**A,B). As the ex vivo images of lungs and their quantitative ROI analysis at 24 h post‐injection suggested, DiR@LRM showed significantly enhanced accumulation in the lung than DiR@Lip and DiR@LR, indicating the great targeting and retention of this hybrid nanocarriers in target organ.

Given its favorable targeting ability towards infected lungs, the therapeutic performance of AB@LRM against the acute bacterial pneumonia infected by *P. aeruginosa* was investigated. The therapeutic effects of AB@LRM were evaluated according to the treatment plan outlined (Figure 6C). Firstly, the photothermal performance of different NPs in vivo was explored to verify their PTT effectiveness in the lung (Figure S22, Supporting Information). The lung temperature in mice injected with AB@LRM NPs could climb to approximately 42.9 °C after 10 min irradiation. On the contrary, the PBS group showed negligible temperature rise under the same irradiation conditions. These results indicated the high PTT efficiency of AB@LRM NPs to treat bacterial pneumonia. Subsequently, the lung tissues were collected at the end of the therapy cycle for further analysis. The lung in PBS group experienced significant pulmonary edema and hemorrhage. Conversely, these phenomena were obviously relieved after treatment with AB@LRM (+) (Figure 6E). In addition, residual bacteria in the lung tissue were evaluated by a spread plate method (Figure 6D). Notably, the number of bacteria in the lungs of AB@LRM (+)‐treated mice decreased significantly. However, obvious bacteria existed in the lung tissues of other treatment groups, indicating that AB@LRM could effectively kill the bacteria in the lung when exposed to NIR irradiation (Figure 6F,H). Besides, bacterial infections in the lungs could induce increased inflammatory secretions, leading to pulmonary edema.^[^ ^41^ ^]^ Therefore, the degree of pulmonary edema was determined by measuring the wet/dry ratio of the lung tissues. When comparing the lung tissue of mice in AB@LRM (+) group to that in PBS group, a significantly lower wet/dry ratio was seen, indicating that the lung edema was effectively alleviated (Figure 6G). Moreover, the survival rate of mice following different treatments was monitored. In the PBS group, the survival rate of mice was only 50%, while it reached 100% in AB@LRM (+) group, demonstrating that treatment using our designed NPs could largely improve the survival of mice suffered from bacterial pneumonia (Figure 6I).

**Figure 7:**
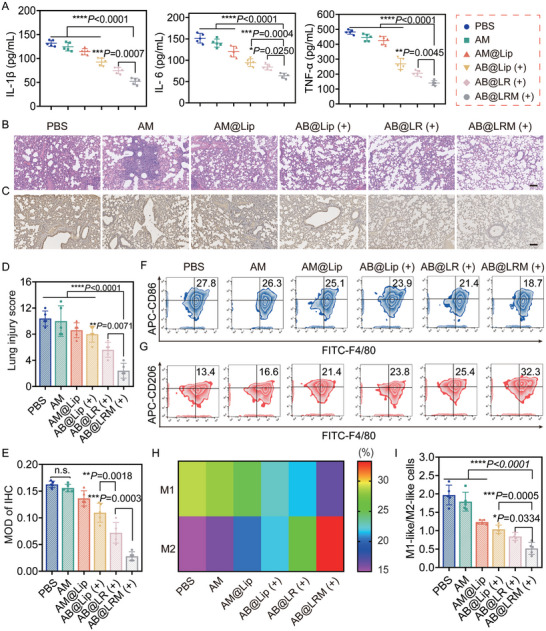
Antibacterial mechanisms of AB@LRM against bacterial pneumonia infected by *P. aeruginosa*. A) Cytokine levels of IL‐1β, IL‐6, TNF‐α in lungs of mice after different treatment groups (*n* = 5). B,D) H&E staining of lungs (scale bar = 100 µm) and quantitative analysis of H&E score (*n* = 5). C,E) IHC staining and quantitative analysis of NF‐κB p65 expression in lungs (scale bar = 100 µm) (*n* = 5). F–H) Representative FCM plots of macrophage subpopulations in the lung after different treatments and their heat map analysis (*n* = 5). I) Quantitative analysis of the ratio of M1‐like macrophages to M2‐like macrophages in the lung after different treatments (*n* = 5). *p < 0.05, **p < 0.01, ***p < 0.001, ****p < 0.0001.

Infection by *P. aeruginosa* dramatically elevated the expressions of inflammatory cytokines such as IL‐1β, IL‐6 and TNF‐α.^[^ ^42^ ^]^ Therefore, lung tissue and serum were collected on day 9 after different treatments and the cytokines were measured using enzyme‐linked immunosorbent assay (ELISA) (**Figure** **7**A, Figure S23, Supporting Information). In PBS group, IL‐1β, IL‐6 and TNF‐α showed high expressions. By contrast, the inflammation was relieved after different treatments in varying degrees, whereas AB@LRM (+) led to the lowest expression levels of these inflammatory cytokines, suggesting its superior anti‐inflammatory activity. Moreover, pathological sections were examined by H&E staining to assess immunological infiltration and lung damage (Figure 7B,D). The alveolar tissue of the PBS and AM groups had severe injuries and an abundance of inflammatory cells. However, in comparison to the other treatment groups, the completeness of the alveoli in the lungs from AB@LRM (+) group was significantly better and the number of inflammatory cells was much lower, which intuitively demonstrated that the inflammation had been relieved and the damage to lung tissue had been well repaired. NF‐κB p65 was invariably upregulated in airway epithelial cells following *P. aeruginosa*‐caused lung infection and the overexpression of proinflammatory cytokines is due to the elevated NF‐κB p65 expression.^[^ ^43^ ^]^ Therefore, the inflammatory response following various treatments was examined by immunohistochemistry (IHC). According to IHC analysis, there was a high expression of NF‐κB p65 in lung tissues in PBS and AM groups, which enhanced the inflammatory response and induced lung injury. However, treatment using AB@LRM (+) significantly reduced NF‐κB p65 expression levels compared to the other treatment groups, indicating that the combination of AB@LRM and NIR irradiation effectively reduced NF‐κB p65 secretion to attenuate the inflammatory response (Figure 7C,E).

Macrophages play an important role in modulating the inflammatory and tissue repair processes. M1‐like macrophages are important pro‐inflammatory immune cells that promote the inflammation process by secreting inflammatory substances during the acute phase of infection, whereas M2‐like macrophages are anti‐inflammatory and promote tissue repair.^[^ ^44^ ^]^ To clarify the antibacterial effect of AB@LRM (+) in bacterial pneumonia, the percentage of macrophages from different types in the lung tissues after various treatments was detected by FCM. According to the FCM results, severe infection occurred in both PBS group and AM group, and the proportion of M1‐like macrophages (CD86^+^ F4/80^+^) also increased significantly. By contrast, AB@LRM (+) group resulted in more infiltrations of M2‐like macrophages (CD206^+^ F4/80^+^) with fewer M1‐like macrophages in the lungs (Figure 7F–I; Figure S24, Supporting Information). The above investigations indicated that treatment using AB@LRM (+) efficaciously weakened infection responses and alleviated local inflammatory symptoms, leading to the remodeling of inflammation‐related macrophages in lung tissues.

### 2.7 Biocompatibility and Toxicity Assessment of AB@LRM

**Figure 8:**
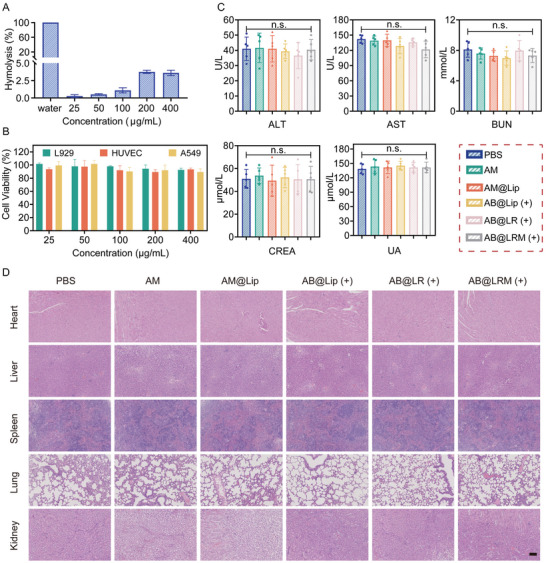
Biocompatibility and toxicity assessment of AB@LRM. A) Hemolytic ratios after treatment with different doses of AB@LRM (*n* = 3). B) Cell viability of L929, HUVEC and A549 cells treated with various concentrations of AB@LRM (*n* = 3). C). Evaluation of liver and kidney function via biomarker analysis of blood serum (*n* = 5). D) Histological images of heart, liver, spleen, lung, and kidney samples from mice after i.v. injection of corresponding drugs (scale bar = 100 µm).

Apart from desired therapeutic effect, the biocompatibility of designed nanocarrier also plays a key role in its in vitro and in vivo application. Therefore, the hemocompatibility of AB@LRM was evaluated using the hemolysis test. No obvious hemolysis of RBC less than 5% was noticed after 1 h incubation of AB@LRM even at its highest concentration of 400 µg mL^−1^, whereas the positive control group that received deionized water displayed significant hemolysis (**Figure** **8**A). In addition, MTT method was employed to assess the cytotoxicity of AB@LRM against different cell lines including L929, HUVECs and A549 cells. When the concentration of AB@LRM was maintained below 400 µg mL^−1^, the cell viability was all over 80%, which suggested that there was no discernible toxicity of AB@LRM in applied dosage in this work (Figure 8B).

To further assess the in vivo toxicity of our designed NPs, six groups of healthy mice were randomly assigned and treated with PBS, AM, AM@Lip, AB@Lip (+), AB@LR (+) and AB@LRM (+) routinely via the tail vein every other day. On the tenth day, blood biochemistry studies were performed, which showed that the kidney function indexes (blood urea nitrogen (BUN), uric acid (UA) and creatinine (CREA)) and liver function indexes (aspartate aminotransferase (AST) and alanine aminotransferase (ALT)) of mice treated with AB@LRM (+) group were all in the normal range, indicating that AB@LRM (+) had no obvious hepatorenal toxicity (Figure 8C).^[^ ^45^ ^]^ H&E staining was performed to analyze the pathological changes in key organs to further examine the toxicity of AB@LRM (+), and we found that there were no inflammatory lesions, histological abnormalities, fibrosis, or necrosis among the six groups (Figure 8D). Moreover, we monitored the daily changes in body weight of mice suffered from bacterial infections both in local wounds and lungs, which displayed that the body weight of mice in all groups maintained within a reasonable range (18–22 g) (Figures S25 and S26, Supporting Information). Therefore, all these results verified the good biocompatibility of AB@LRM (+) with no significant damage to major organs and blood parameters.

## 3 Conclusion

In summary, we designed a multifunctional and biomimetic nanoplatform with excellent inflammation targetability and long circulation ability for synergistic antibacterial therapies. Specifically, RBC membrane and macrophage membrane were fused to obtain hybrid cell membrane coating, which was clothed around drug‐loaded liposomes to co‐deliver classical antibiotics AM and photosensitizer BPQDs for combinational therapeutic purpose. The designed AB@LRM inherited the long circulation property of RBC and the bacterial targeting ability of macrophage, which achieved accurate drug delivery to the infected locations. Importantly, BPQDs not only produced ROS, but also showed good thermal effect on bacteria when exposed to NIR irradiation, eventually enabling bacteria to become more sensitive to antibiotics. Therefore, the favorable PTT and PDT performance produced by AB@LRM (+) efficiently synergized with chemotherapy to achieve an improved sterilizing effect, which also significantly reduced the dosage of antibiotics, thereby decreasing the possibility of bacterial resistance. By validating the therapeutic efficacy of AB@LRM (+) in vitro and in vivo based on wound infection and acute pneumonia infection, two typical infection models of *P. aeruginosa*, we found that AB@LRM (+) displayed potent antibacterial ability both in vitro and in vivo, which not only effectively killed bacteria at a low dosage, but also relieved the inflammatory environment of bacterial infections, promoted wound healing, and reduced the damage caused by bacterial pneumonia. Overall, AB@LRM (+) designed in this work exhibited great potentials in the fields of antibacterial therapy and could be developed as a safe and effective nanotool to treat bacterial infections.

## 4 Experimental Section

Materials, methods, preparation, and characterization of AB@LRM NPs, in vitro and in vivo antibacterial studies and in vitro and in vivo antibacterial mechanisms are provided in Supporting Information.

### Animal Experiment

All animal procedures were performed under the National Institute of Health Guidelines for the Care and Use of Laboratory Animals and approved by the Ethics Committee of China Pharmaceutical University (Ethics Code: 2022‐05‐044).

### Statistical Analysis

Each experiment was repeated at least three times and all data were shown as means ± SD. Statistical significance was determined by unpaired two‐tailed Student's t‐tests where only two groups existed or by one‐way ANOVA to compare three or more groups. Differences between groups were considered significant at p < 0.05. (*p < 0.05, **p < 0.01, ***p < 0.001, ****p < 0.0001).

## Conflict of Interest

The authors declare no conflict of interest.

## Author Contributions

H.L. and L.T. contributed equally to this work. W.W., Y.S., H.L., and L.T. conceived and designed the experiments. H.L., L.T., Y.Y., Y.C., C.F., and J.F. performed the experiments. H.L. and L.T. analyzed the data. H.L., L.T., Y.S., and W.W. wrote and edited the manuscript. W.W., Y.S., H.L., and L.T. were involved in the discussion. W.W. and Y.S. supervised the entire project. All authors discussed the results and commented on the manuscript.

## Supporting information

Supporting Information

## Data Availability

The data that support the findings of this study are available from the corresponding author upon reasonable request.

## References

[1] a) W. Xie , J. Chen , X. Cheng , H. Feng , X. Zhang , Z. Zhu , S. Dong , Q. Wan , X. Pei , J. Wang , Small 2023, 19, e2205941; 36587967 10.1002/smll.202205941

[2] C. Wang , Y. Lin , J. Huang , H. Song , Y. Zhang , Y. Zhang , M. Xu , J. Liu , Nano Today 2023, 51, 101892.

[3] X. He , L. Dai , L. Ye , X. Sun , O. Enoch , R. Hu , X. Zan , F. Lin , J. Shen , Adv. Sci. 2022, 9, e2105223. 10.1002/advs.202105223 PMC9108595 35274475

[4] a) H. Yuan , X. Hong , H. Ma , C. Fu , Y. Guan , W. Huang , J. Ma , P. Xia , M. Cao , L. Zheng , X. Xu , C. Xu , D. Liu , Z. Li , Q. Geng , J. Wang , ACS Mater. Lett. 2023, 5, 762;

[5] C. Zhang , J. Wu , W. Liu , W. Zhang , C.‐S. Lee , P. Wang , Acta Biomater. 2023, 159, 247. 36724864 10.1016/j.actbio.2023.01.031

[6] a) X. Yu , J. Zhao , D. Fan , Chem. Eng. J. 2022, 437, 135475;

[7] a) L. He , D. Di , X. Chu , X. Liu , Z. Wang , J. Lu , S. Wang , Q. Zhao , J. Controlled Release 2023, 363, 180; 10.1016/j.jconrel.2023.09.035 37739014

[8] G. Qing , X. Zhao , N. Gong , J. Chen , X. Li , Y. Gan , Y. Wang , Z. Zhang , Y. Zhang , W. Guo , Y. Luo , X. J. Liang , Nat. Commun. 2019, 10, 4336. 31551496 10.1038/s41467-019-12313-3 PMC6760232

[9] K. Zhu , S. Qian , H. Guo , Q. Wang , X. Chu , X. Wang , S. Lu , Y. Peng , Y. Guo , Z. Zhu , T. Qin , B. Liu , Y. W. Yang , B. Wang , ACS Nano 2022, 16, 11136. 35749223 10.1021/acsnano.2c03971

[10] a) S. Geng , Z. Li , R. Zhang , W. Zhou , G. Luo , P. K. Chu , X.‐F. Yu , Chem. Eng. J. 2021, 421, 127879;

[11] L. Tang , J. Li , T. Pan , Y. Yin , Y. Mei , Q. Xiao , R. Wang , Z. Yan , W. Wang , Theranostics 2022, 12, 2290. 35265211 10.7150/thno.69628 PMC8899561

[12] a) X. Gui , H. Zhang , R. Zhang , Q. Li , W. Zhu , Z. Nie , J. Zhao , X. Cui , W. Hao , X. Wen , W. Shen , H. Song , Mater. Today Bio 2023, 19, 100602; 10.1016/j.mtbio.2023.100602 PMC10024194 36942311

[13] J. Fu , Y. Li , Y. Zhang , Y. Liang , Y. Zheng , Z. Li , S. Zhu , C. Li , Z. Cui , S. Wu , Adv. Mater. 2021, 33, 2102926. 10.1002/adma.202102926 34396595

[14] L. Ran , B. Lu , H. Qiu , G. Zhou , J. Jiang , E. Hu , F. Dai , G. Lan , Bioact. Mater. 2021, 6, 2956. 33732966 10.1016/j.bioactmat.2021.01.032 PMC7930507

[15] a) C. Gu , S. Yang , X. Liu , Y. Jin , Y. Yu , L. Lu , Nano Res. 2023, 16, 11401;

[16] a) W. Wang , Y. Gao , M. Zhang , Y. Li , B. Z. Tang , ACS Nano 2023, 17, 7394; 37009988 10.1021/acsnano.2c11762

[17] P. H. D. Nguyen , M. K. Jayasinghe , A. H. Le , B. Peng , M. T. N. Le , ACS Nano 2023, 17, 5187. 36896898 10.1021/acsnano.2c11965

[18] a) X. Qin , L. Zhu , Y. Zhong , Y. Wang , G. Wu , J. Qiu , G. Wang , K. Qu , K. Zhang , W. Wu , Adv. Sci. 2023, 10, 2205093; 10.1002/advs.202205093 PMC9951580 36703487

[19] a) H. Cao , Y. Gao , H. Jia , L. Zhang , J. Liu , G. Mu , H. Gui , Y. Wang , C. Yang , J. Liu , Nano Lett. 2022, 22, 7882; 36169350 10.1021/acs.nanolett.2c02560

[20] a) H. Hu , S. Y. Hua , X. Lin , F. Lu , W. Zhang , L. Zhou , J. Cui , R. Wang , J. Xia , F. Xu , X. Chen , M. Zhou , ACS Nano 2023, 17, 11692; 37310363 10.1021/acsnano.3c02365

[21] S. Huang , S. Xu , Y. Hu , X. Zhao , L. Chang , Z. Chen , X. Mei , Acta Biomater. 2022, 137, 199. 34644613 10.1016/j.actbio.2021.10.008

[22] D. Wang , H. Dong , M. Li , Y. Cao , F. Yang , K. Zhang , W. Dai , C. Wang , X. Zhang , ACS Nano 2018, 12, 5241. 29800517 10.1021/acsnano.7b08355

[23] a) P. You , A. Mayier , H. Zhou , A. Yang , J. Fan , S. Ma , B. Liu , Y. Jiang , Appl. Mater. Today 2022, 26, 101386;

[24] L. Chen , Z. Shao , Z. Zhang , W. Teng , H. Mou , X. Jin , S. Wei , Z. Wang , Y. Eloy , W. Zhang , H. Zhou , M. Yao , S. Zhao , X. Chai , F. Wang , K. Xu , J. Xu , Z. Ye , Adv. Mater. 2024, 36, 2304774. 10.1002/adma.202304774 37523329

[25] Z. Guo , Y. Zhu , G. Du , M. Qin , C. He , P. He , Y. Song , W. Chen , S. Bai , F. Wu , N. Qiao , M. Jiang , X. Luo , Y. Zhang , T. Gong , Z. Zhang , X. Sun , Nano Today 2022, 43, 101398.

[26] X. Zhao , J. Feng , J. Zhang , Z. Han , Y. Hu , H. H. Shao , T. Li , J. Xia , K. Lei , W. Wang , F. Lai , Y. Lin , B. Liu , K. Zhang , C. Zhang , Q. Yang , X. Luo , H. Zhang , C. Li , W. Zhang , S. Wu , Acta Pharm. Sin. B 2023, 13, 4945. 38045053 10.1016/j.apsb.2023.08.030 PMC10692473

[27] a) Y. Dai , J. Mei , Z. Li , L. Kong , W. Zhu , Q. Li , K. Wu , Y. Huang , X. Shang , C. Zhu , Small 2022, 18, 2204377; 10.1002/smll.202204377 36216771

[28] J. Cheng , G. Gan , S. Zheng , G. Zhang , C. Zhu , S. Liu , J. Hu , Nat. Commun. 2023, 14, 7510. 37980361 10.1038/s41467-023-43415-8 PMC10657346

[29] L. Tang , S. He , Y. Yin , H. Liu , J. Hu , J. Cheng , W. Wang , Pharmaceutics 2021, 13, 1888. 34834304 10.3390/pharmaceutics13111888 PMC8621332

[30] L. Tang , Y. Yin , Y. Cao , C. Fu , H. Liu , J. Feng , W. Wang , X. J. Liang , Adv. Mater. 2023, 35, 2303835. 10.1002/adma.202303835 37384818

[31] a) N. Li , G. Wu , L. Tang , W. Zhou , S. Yang , Q. Pan , M. Wang , P. Wu , H. Xiao , Y. He , X. Tan , Q. Yang , ACS Appl. Mater. Interfaces 2022, 14, 46362; 36198018 10.1021/acsami.2c15759

[32] Y. H. Hsieh , W. C. Chuang , K. H. Yu , C. P. Jheng , C. I. Lee , Pharmaceutics 2019, 11, 16. 30621174 10.3390/pharmaceutics11010016 PMC6359070

[33] G. Niu , F. Gao , C. Li , Y. Wang , H. Li , Y. Jiang , J. Mater. Chem. B 2023, 11, 8916. 37545365 10.1039/d3tb01376f

[34] C. Tong , X. Zhong , Y. Yang , X. Liu , G. Zhong , C. Xiao , B. Liu , W. Wang , X. Yang , Biomaterials 2020, 243, 119936. 32171103 10.1016/j.biomaterials.2020.119936

[35] Y. Chen , X. Wang , S. Tao , Q. Wang , P. Q. Ma , Z. B. Li , Y. L. Wu , D. W. Li , Mil. Med. Res. 2023, 10, 37. 37608335 10.1186/s40779-023-00473-9 PMC10463485

[36] Y. Zhang , C. Wen , Y. Liu , A. Li , Q. Guo , X. Zhang , L. Fu , S. Xu , D. Qiao , P. Zheng , W. Zhu , Q. Pan , Chem. Eng. J. 2023, 470, 144084.

[37] a) Y. Long , L. Li , T. Xu , X. Wu , Y. Gao , J. Huang , C. He , T. Ma , L. Ma , C. Cheng , C. Zhao , Nat. Commun. 2021, 12, 6143; 34686676 10.1038/s41467-021-26456-9 PMC8536674

[38] Z. Wang , J. Lu , Z. Yuan , W. Pi , X. Huang , X. Lin , Y. Zhang , H. Lei , P. Wang , Small 2023, 19, 2205528. 10.1002/smll.202205528 36446719

[39] Z. Wang , H. Lu , T. Tang , L. Liu , B. Pan , J. Chen , D. Cheng , X. Cai , Y. Sun , F. Zhu , S. Zhu , Cell Proliferation 2022, 55, e13316. 35869570 10.1111/cpr.13316 PMC9628242

[40] Y. Tian , Y. Li , J. Liu , Y. Lin , J. Jiao , B. Chen , W. Wang , S. Wu , C. Li , Bioact. Mater. 2022, 9, 428. 34820581 10.1016/j.bioactmat.2021.07.033 PMC8586811

[41] P. Liu , P. Ji , L. Wang , H. Guo , M. Huo , J. Shi , Biomaterials 2022, 289, 121768. 36088676 10.1016/j.biomaterials.2022.121768

[42] Q. Guo , Y. Luo , H. Guo , T. Lan , S. Wang , K. Geng , X. Lu , L. Tao , X. Shen , Chem. Eng. J. 2022, 446, 137173.

[43] S. Mu , Y. Zhu , Y. Wang , S. Qu , Y. Huang , L. Zheng , S. Duan , B. Yu , M. Qin , F. J. Xu , Adv. Mater. 2022, 34, 2204065. 10.1002/adma.202204065 35962720

[44] H. Tang , X. Qu , W. Zhang , X. Chen , S. Zhang , Y. Xu , H. Yang , Y. Wang , J. Yang , W. E. Yuan , B. Yue , Adv. Mater. 2022, 34, 2107300. 10.1002/adma.202107300 34865257

[45] M. Lu , H. Xing , W. Shao , T. Zhang , M. Zhang , Y. Wang , F. Li , Y. Weng , A. Zheng , Y. Huang , X. J. Liang , Adv. Mater. 2022, 34, 2204765. 10.1002/adma.202204765 35793475

