# Supplementary material for: Photoresponsive Multirole Nanoweapon Camouflaged by Hybrid Cell Membrane Vesicles for Efficient Antibacterial Therapy of Pseudomonas aeruginosa‐Infected Pneumonia and Wound
Source: Adv Sci (Weinh). 2024 Jul 15;11(35):2403101. doi: 10.1002/advs.202403101 (PMC11425291; doi:10.1002/advs.202403101)
Supplement: Supplementary file 1 — Supporting Information [file ADVS-11-2403101-s001.pdf]

Supporting Information

Photo-Responsive Multi-Role Nanoweapon Camouflaged by Hybrid Cell Membrane Vesicles for Efficient Antibacterial Therapy of *Pseudomonas aeruginosa*-Infected Pneumonia and Wound

Hening Liu, Lu Tang, Yue Yin, Yuqi Cao, Cong Fu, Jingwen Feng, Yan Shen, and Wei Wang**

H. Liu, L. Tang, Y. Yin, Y. Cao, C. Fu, J. Feng, Y. Shen, and W. Wang

State Key Laboratory of Natural Medicines, Department of Pharmaceutics, School of Pharmacy, China Pharmaceutical University, Nanjing 211198, P. R. China

E-mail: shenyan@cpu.edu.cn (Y. Shen); wangcpu209@cpu.edu.cn (W. Wang)

H. Liu, L. Tang, Y. Yin, Y. Cao, C. Fu, J. Feng, and W. Wang

NMPA Key Laboratory for Research and Evaluation of Cosmetics, China Pharmaceutical University, Nanjing 211198, P. R. China

Email: wangcpu209@cpu.edu.cn (W. Wang)

Experimental Section

Materials

Amikacin (AM), cholesterol and sodium 1-octanesulfonate monohydrate were purchased from Shanghai Macklin Biochemical Co., Ltd. (Shanghai, China). Bulk black phosphorus (BP) was purchased from Kunming Black Phosphorus Technology Service Co., Ltd. (Kunming, China). Coumarin-6 (C6), N-methyl-2-pyrrolidone (NMP) and 3-(4, 5-dimethylthiazol-2-yl)-2, 5- diphenyltetrazolium bromide (MTT) were purchased from Shanghai Aladdin Biochemical Technology Co., Ltd. (Shanghai, China). DOTAP and DOPE were purchased from A.V.T. Pharmaceutical Co., Ltd. (Shanghai, China). FITC, DiR, DiD and DiI were purchased from Yuanye Biological Technology Co., Ltd. (Shanghai, China). Phosphate buffer saline (PBS), fetal bovine serum (FBS), RPMI-1640 medium, DMEM medium, and Penicillin-Streptomycin liquid were obtained from SenBeiJia Biological Technology Co., Ltd. (Nanjing, China). Luria-Bertani (LB) medium and agar were purchased from Guangdong Huankai Microbial Sci.&Tech. Co., Ltd. (Guangdong, China). 4',6-diamidino-2-phenylindole (DAPI), enhanced BCA protein assay kits and ROS assay kit were purchased from Beyotime Biotechnology Co., Ltd. (Shanghai, China). Live/Dead bacterial double stain kit was purchased from Shanghai Kanglang Biotechnology Co., Ltd. (Shanghai, China). ATP detection kit, anti-F4/80-FITC antibody, anti-CD86-APC antibody and anti-CD206-APC antibody were purchased from Elabscience Biotechnology Co., Ltd. (Wuhan, China).

Methods

Preparation of BPQDs

The black phosphorus quantum dots (BPQDs) were obtained by a modified liquid exfoliation method.[1] Briefly, bulk black phosphorus (BP, 20.0 mg) was dispersed in N-Methylpyrrolidone (NMP, 20 mL) and ground for 5 min. Then, an ultrasonic cell disruption

system (JY92-IIDN, Scientz, China) was used to sonicate the mixture in an ice bath for 6 h (800 W), which was followed by 10 h (200 W) of ultrasonication in water bath (BD-5200DT, Scientz, China). The resultant mixture of BPQDs was centrifuged at 1000 rpm for 15 min to remove large particles. To further remove NMP, the supernatant was centrifuged for 15 min at 15,000 rpm. (L-80XP, Beckman, USA). Finally, the precipitate was prepared BPQDs and was dispersed in water.

Preparation of AB@Lip nanoparticles

AB@Lip nanoparticles (NPs) were synthesized using the previously reported method of film dispersion method.[2] Briefly, 2.5 mg DOTAP, 2.5 mg DOPE and 1.25 mg cholesterol were dissolved in 5 mL of methanol and the solvent was then evaporated to create a film in the round-bottomed flask by means of a rotary evaporator (RE-52S, Yarong, China). Then, the resultant lipid film was hydrated with 5 mL of solution containing 1 mg BPQDs and 1.2 mg AM before sonication (300 W, 15 min). Finally, the homogeneous liposome suspension was obtained by passing through membrane filters.

Extraction of mouse red blood cell membrane

The red blood cell membrane (Rm) was extracted from whole blood of mice in accordance with previously reported protocols.[3] In brief, whole blood was centrifuged at 800×g for 10 min. Then, the red blood cell (RBC) precipitate was cleaned with PBS and resuspended in cold hypotonic lysing buffer (0.25 × PBS) for 30 min. The lysed RBC was centrifuged at 2000 g for 15 min to remove the impurities. Finally, the supernatant was centrifuged at 10000 g for 1 h. The Rm was gathered and kept in storage at -80 °C.

Extraction of macrophage membrane

Collected macrophages were suspended in precooled tris-magnesium (TM) buffer at a density of 2.5 × 10^7 cells/mL and placed at 4 °C for 12 h.[4] Cells were broken using a sonicator for 10 min. The homogenate was added with 0.25 M (final concentration) sucrose before centrifugation for 10 min at 2000 g. The supernatant was gathered and then centrifuged for 30 min at 3000 g to extract the macrophage membrane (Mm). Finally, Mm was centrifuged at 3000 g for 30 min and rinsed in TM buffer containing 0.25 M sucrose and kept in storage at -80 °C.

Preparation of AB@LRM NPs

The BCA protein assay was used to quantify the total protein concentration of Rm and Mm. Afterward, the Rm solution was equally added to the Mm solution and sonicated (100 W) at 37°C for 5 min to obtain the red blood cell-macrophage hybrid membrane (RM). Subsequently, RM was mixed with AB@Lip and extruded 15 times using the liposome extruder (Avestin, Canada) to create AB@LRM.

Characterization of AB@LRM NPs

The zeta potential and hydrodynamic diameter were determined using dynamic light scattering (DLS, Nano ZS90, Malvern, UK). To examine the morphology of samples, transmission electron microscopy (TEM, Hitachi, Japan, 100 kV) was employed. Rm and Mm were labeled with DiD and DiI, respectively. AB@Lip NPs were labeled with FITC. AB@LRM NPs were prepared according to the fusion method described above and observed using confocal laser scanning microscopy (CLSM, Zeiss LSM700, Germany). To examine the total proteins of AB@LRM, sodium dodecyl sulfate-polyacrylamide gel electrophoresis (SDS-PAGE) was used. Western blotting analysis was further applied to determine the key

proteins on AB@LRM, such as TLR4 and CD47. Additionally, the membrane fusion efficiency of AB@LRM NPs was evaluated by flow cytometry (FCM, Celesta, BD, USA).

Förster resonance energy transfer study

The membrane fusion process was observed using the Förster resonance energy transfer (FRET) method.[5] Briefly, Mm solution was mixed with DiI (excitation/emission = 549/565nm) and DiD (excitation/emission = 644/663 nm) and stirred at 37°C for 1 h. Subsequently, the mixture was centrifuged at 13000 rpm for 15 min to remove free dyes. Rm was incorporated into a solution of Mm at various membrane protein weight ratios (0:1, 1:1, 3:1, and 5:1), and complete membrane fusion was achieved by sonicating the mixture for 10 min. Then, at an excitation wavelength of 525 nm, the fluorescence spectra of all samples were investigated between 550 and 750 nm. In order to demonstrate the alteration in FRET efficiency, fluorescence intensities of DiD (663 nm) and DiI (565 nm) were recorded. The optical properties of AB@LRM were measured with a fluorescence spectrophotometer (RF5301PC, Shimadzu, Japan) with a 1 cm cuvette.

Photothermal effect and photothermal stability of AB@LRM NPs

The photothermal performance of AB@LRM was verified by monitoring temperature changes of various concentrations of AB@LRM (BPQDs content: 0~50 μg/mL) under 808 nm NIR laser irradiation with various power densities (0.5, 1.0, 1.5 and 2 W/cm ^{2}) for 10 min. Moreover, BPQDs, AB@Lip or AB@LRM solution were irradiated at 1.5 /W cm^2 for 10 min. An infrared thermal camera (H10, Hikvision, China) was used to capture the real-time thermal images and the temperature change of the dispersion liquid. To examine the photothermal stability of AB@LRM, the above dispersion was placed in exposed air for 5 days, and the temperature changes and infrared thermal images were recorded under an NIR laser

irradiation (1.5 W/cm^2) for 10 min. Finally, the recycling heating–cooling curve of AB@LRM (50 μg/mL) with NIR irradiation (1.5 W/cm^2) was recorded for five on/off cycles.

Release of AM

Drug release assay was performed at pH 5.5 and pH 7.4 to obtain the release profiles of AM from the AB@LRM. In brief, a dialysis bag (MWCO 3500 Da) containing 1 mL of NPs was incubated in 20 mL of PBS at pH 5.5 and pH 7.4, separately. Subsequently, 1 mL of the dialysis solution was removed and replaced with 1 mL of PBS at 1, 2, 4, 6, 8, 10, 12, 24, 36, and 48 h. Finally, high-performance liquid chromatography (HPLC, LC-20AT, Shimadzu, Japan, C18 column, 40 °C) was used to quantify the released AM. 1.8 g of sodium octane sulfonate and 20.0 g of anhydrous sodium sulfate were mixed with 0.2 mol/L phosphate buffer (pH 3.0, 50 mL), water (875 mL) and acetonitrile (75 mL), which was used as the mobile phase. The flow rate was 1.3 mL/min and the detection wavelength was 200 nm.

Bacterial culture

Pseudomonas aeruginosa (*P. aeruginosa*) (ATCC 27853) was purchased from Shanghai Shifeng Biotechnology Co., Ltd. (Shanghai, China). *P. aeruginosa* was cultured using LB medium and shaken during incubation at 37 °C for 8 h (THZ-Q, Huamei, China). Before the antibacterial experiment, PBS was used to adjust the optical density at 600 nm (OD_{600}) of *P. aeruginosa* solution to 1 for further use, and the concentration of *P. aeruginosa* was 10^9 CFU/mL at this time.

Determination of minimum inhibitory concentration

The micro-broth dilution method was used to obtain the minimum inhibitory concentrations (MIC) of AM and AB@LRM (+) against *P. aeruginosa*. Bacteria in the logarithmic growth

phase were diluted with LB medium to 1×10^6 CFU/mL and placed on the 96-well plate. To measure MIC, diluted samples (100 μL) in a series of AM concentrations were added into corresponding wells. For the laser treatment group, the NIR irradiation (808 nm, 1.5 W/cm^2, 10 min) was applied. Meanwhile, LB medium served as the blank control, and bacteria in LB medium were employed as the positive control. A microplate reader was used to determine the OD_{600} value of bacterial suspension after 18 h of incubation at 37 °C.

Growth curve observation

After reaching the logarithmic phase, the bacteria were diluted to approximately 10^6 CFU/ mL with the culture medium and treated with various prepared samples respectively. After incubation for 4 h, each group was treated without or with NIR irradiation. Subsequently, 100 μL of bacterial suspension from each sample was added to 96-well plates and continuously incubated at 37 °C. The OD_{600} value of all the wells was recorded at 600 nm every 2 h for 24 h to observe *P. aeruginosa* growth curves.

Observation of the colony-forming units in vitro

The antibacterial property of AB@LRM was measured on an agar plate by counting colony-forming units (CFU). In short, the bacterial suspensions in the mid-log growth phase were diluted to 1.0 × 10^6 CFU/mL and were treated with AM, BPQDs, AB, AB@Lip and AB@LRM, and the PBS group served as the blank control. After 4 h of co-incubation, NIR irradiation groups were treated with an 808 nm laser. Thereafter, all the groups treated for 24 h at 37 °C were diluted with PBS solutions and 100 μL of the bacterial suspensions were spread onto LB plates. After incubation for 18 h at 37 °C, the number of colonies was counted and photographed.

Biofilm dispersion assay

Firstly, a biofilm was created by adding 200 μL of *P. aeruginosa* (5×10^8 CFU/mL) to 96-well plates and incubated at 37 °C. After 48 h, 100 μL of normal PBS, AM, BPQDs, AB@Lip and AB@LRM were added into the wells and cultured for 12 h. The same procedure as described above was used for the laser treatment groups. Subsequently, the biofilm was fixed with 200 μL methanol and cultured for 30 min at room temperature. After fixation, each well received 200 μL of 0.1% crystal violet to stain the biofilm for 30 min and then washed three times with PBS. Lastly, each well was added with 200 μL of 95% ethanol to dissolve the dye and the biofilm biomass was quantified using a microplate reader at 590 nm.

Live/dead bacterial staining assay

To evaluate bacteria viability, a Live/Dead kit that uses PI to label dead bacteria and SYTO 9 to label live bacteria was employed. Specifically, bacteria were incubated with PBS (control group), AM, BPQDs (+), AB (+), AB@Lip (+) or AB@LRM (+) at 1 μg/mL of AM for 24 h. Differently treated *P. aeruginosa* were mixed with fluorescent dyes and incubated in dark for 30 min. Afterward, treated samples were washed with PBS and placed on a glass slide. Dead (red fluorescence) bacteria and live (green fluorescence) bacteria were distinguished using CLSM, and Image J software was used to quantitatively analyze both types of bacteria.

Uptake of NPs by macrophages

The immune escape ability of the prepared NPs was determined using RAW264.7 cells. In brief, cells were inoculated into the 24-well plate (5×10^4 cells/well) and cultured for 24 h. Next, C6-labeled AB@Lip and AB@LRM were added and incubated. After 5 h, macrophages were washed with PBS and then stained with DAPI. The fluorescence images of macrophages were obtained using an inverted fluorescence microscope (IFM, Olympus IX53).

Detection of bacterial ROS production

A 2', 7'- dichlorodihydrofluorescein diacetate (DCFH-DA) probe was used to measure the intracellular ROS concentration. In summary, bacteria were cultured with different prepared NPs for 3 h. Next, bacteria were exposed to 808 nm laser for 10 min before incubation for 1 h. After centrifugation, treated bacteria were incubated with 200 μL of DCFH-DA solution (10 μM) in the dark for 30 min. After that, samples were immediately visualized using a fluorescent inverted microscope to obtain bacterial images. Fluorescence intensity was quantified using Image J software.

Bacterial morphological characterization

A scanning electron microscope (SEM) was used to investigate the alterations of the bacterial morphology. First, the bacterial suspensions of all groups were obtained through centrifugation and washed with PBS. Subsequently, the sample was quickly fixed with 2.5% glutaraldehyde at room temperature for 12 h. Afterwards, the fixed samples were dehydrated with ethanol at various concentrations for 20 min, including 30%, 50%, 70%, 80%, 90%, and 100%.[6] After drying with a vacuum freeze dryer for at least 24 h, the obtained samples were observed via SEM (Phenom PURE+, Phenom, Netherlands, 10 kV).

Adhesion and uptake of NPs by bacteria

Bacteria (1 mL) were cultured with DiI-labeled samples (1 mL) for 1 h, 2 h and 4 h, separately. Subsequently, suspensions were washed with PBS after centrifugation. The obtained bacteria were observed by CLSM after fixed with 4% paraformaldehyde.

ATP test

The ATP kit was used to measure the metabolic activity within *P. aeruginosa*. Briefly, after different treatments mentioned above, *P. aeruginosa* were mixed with 300 μL of lysis buffer.

The sample was placed in a water bath of boiling water for 15 min, then centrifuged at 4 °C, 1000 × g for 15 min. Finally, the gathered supernatant was detected by the multi-mode detection platform under the luminance mode (BioTek Cytation5).

Protein leakage study

In order to quantify the amount of protein released from different samples, bacteria were first washed with PBS to remove the secreted protein, and then treated with various samples. Subsequently, the supernatant was obtained by centrifugation (12000 rpm, 5 min, 4 °C) and the protein concentration was calculated by an enhanced BCA protein assay kit.[7]

Animal

Eight-week-old ICR mice (female, SPF grade) supplied by the Comparative Medicine Center of Yangzhou University (Yangzhou, China) were used in this work. All animal procedures were performed under the National Institute of Health Guidelines for the Care and Use of Laboratory Animals and approved by the Ethics Committee of China Pharmaceutical University (Ethics Code: 2022-05-044).

In vivo synergistic therapeutic effect against wound infection

The effect of AB@LRM (+) on wound healing was evaluated on a *P. aeruginosa*-infected wound model. A circular cutaneous wound with around 8 mm diameter was built on the skin of ICR mice, and then *P. aeruginosa* suspension (20 μL, 1×10^9 CFU/mL) was added to the created wound. After 24 h, mice were randomly divided into six groups including PBS, AM, AM@Lip, AB@Lip (+), AB@LR (+) and AB@LRM (+) groups and received intravenous (*i.v.*) injections once every two days. The concentration of AM in all treatment groups was 4.5 mg/kg. 24 h after *i.v.* injection, the laser treatment groups were irradiated with 808 nm laser (1.5 W/cm^2,10 min) and the temperature of the wound area was captured using an infrared

thermal camera. The wound area and body weight were monitored daily throughout the entire therapy period. After 11 days, infected tissues in all groups were also collected for immunofluorescence (IF) analysis, hematoxylin and eosin (H&E) staining and quantitative evaluation of the bacterial burden using a standard plate counting method.

RNA sequencing of the wound tissue on the skin

For the RNA sequencing (RNA-seq) assay, mice from the healthy group, PBS group and AB@LRM (+) group were sacrificed after 11 days. Skin samples of each group were gathered and placed in liquid nitrogen. Total RNA was extracted from the tissue using TRIzol reagent (Invitrogen, USA). RNA-seq results were analyzed by Dr. Tom Multi-Omics Data Mining System (https://biosys.bgi.com). The Kyoto Encyclopedia of Genes and Genomes (KEGG) database was applied to assess the major pathways with differential genes between various samples (https://www.kegg.jp/). The Gene Ontology (GO) database (http://www.geneontology.org/) was employed to identify differentially expressed genes enriched in the major function.

In vivo biodistribution study

To create a bacterial pneumonia mice model, ICR mice were anesthetized and infected with *P. aeruginosa* (10^8 CFU/mL, 20 μL) by intratracheal injection. After 24 h, the bacteria-infected mice were *i.v.* injected with DiR@Lip, DiR@LR, or DiR@LRM. The dose in each group was equivalent to 1 μg of DiR per mouse. After 24 h, major organs were acquired to observe their fluorescent signals using an imaging system (IVIS Lumina III, Perkin Elmer, USA)

In vivo synergistic therapeutic against bacterial pneumonia

All infected mice were randomly divided into six groups including PBS, AM, AM@Lip, AB@Lip (+), AB@LR (+) and AB@LRM (+) groups and intravenously injected once every

two days. 24 h after *i.v.* injection, the laser treatment groups were exposed with 808 nm laser (1.5 W/cm^2,10 min) and the temperature of infection area was captured using the infrared thermal camera. The weight and survival rate of mice were recorded daily. After 9 days, the serum and lung tissue were collected from each group to measure the levels of IL-1β, TNF-α, and IL-6 using ELISA kits. Wet/dry weight ratios were determined for pulmonary edema evaluation by weighing lung samples both before and after 72 h of drying under 80 °C. As for histological analysis, sliced lung tissues were stained with H&E staining and immunohistochemical (IHC) assay. By distributing lung homogenate onto LB agar plates, bacteria colonies in lung tissue were counted. Image J software was used to quantify the colonies of the leftover bacteria.

FCM assay in vivo

The harvested lung tissues in each treatment group were ground to prepare the single-cell suspensions. Cells were treated with 2 mL of prepared 1× RBC lysis solution for 5 min. Then, collected cells were resuspended with PBS buffer and the cell concentration was adjusted to 1×10^7 cells/mL. Subsequently, macrophages in the lungs were stained with anti-F4/80-FITC antibody, anti-CD86-APC antibody or anti-CD206-APC antibody for 30 min in the dark according to the manufacturer's instructions. After staining, cells were resuspended with 200 μL PBS and detected by FCM. Data analysis was carried out using FlowJo software.

In vivo biosafety assessment

Firstly, the MTT test was used to assess the cytotoxicity of AB@LRM. L929, HUVEC and A549 cells were added into a 96-well plate at a density of 5×10^4 per well for 24 h. Subsequently, AB@LRM was added to the cell culture medium in different concentrations. After 24 h of incubation, 20 μL of MTT solution (5 mg/mL) was added to each well and

incubated for 4 h. Then, 100 μL of DMSO was added to dissolve formazan crystals. Lastly, the absorbance of each well was determined at 490 nm.

Secondly, fresh blood from ICR mice was used for hemolysis determination. 10 mL of blood containing heparin sodium were centrifuged at 2000 rpm for 10 min to remove serum and obtain fresh RBC. Subsequently, RBC was diluted with PBS to create the 2% RBC suspension. Next, 500 μL of RBC suspension was mixed with 1mL of AB@LRM at various concentrations. Deionized water and the 0.9% NaCl solution were utilized as positive and negative controls, accordingly. Each group was incubated at 37 °C for 1 h before, and the absorbance of the supernatants was measured at 540 nm after centrifugation.

Finally, six groups of healthy mice were randomly assigned and treated with PBS, AM, AM@Lip, AB@Lip (+), AB@LR (+) and AB@LRM (+) via the tail vein every other day. Serum was collected for biochemical index detection (blood urea nitrogen (BUN), uric acid (UA), creatinine (CREA), aspartate aminotransferase (AST) and alanine aminotransferase (ALT)) after 10 days. H&E staining was also performed on the primary organs.

Statistical analysis

Each experiment was repeated at least three times and all data were shown as means ± SD. Statistical significance was determined by unpaired two-tailed Student's t-tests where only two groups existed or by one-way ANOVA for comparison of three or more groups. Differences between groups were considered significant at $p < 0.05$. (*$p < 0.05$, **$p < 0.01$, ***$p < 0.001$, ****$p < 0.0001$)

Supporting figures

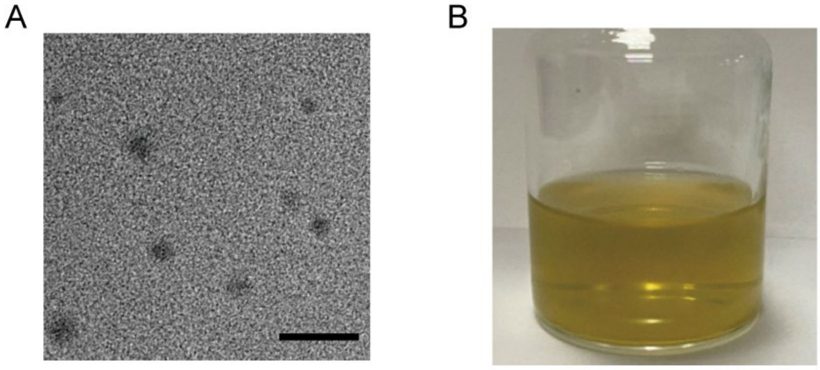

Figure S1. A) TEM image of BPQDs (scale bar = 50 nm). B) Representative photograph of BPQDs suspension in water.

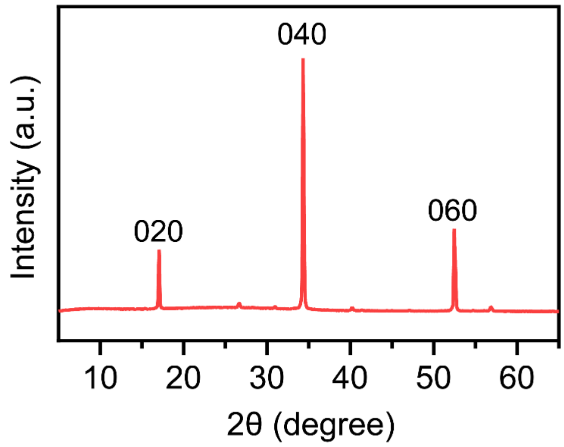

Figure S2. The X-ray diffraction (XRD) spectrum of BPQDs.

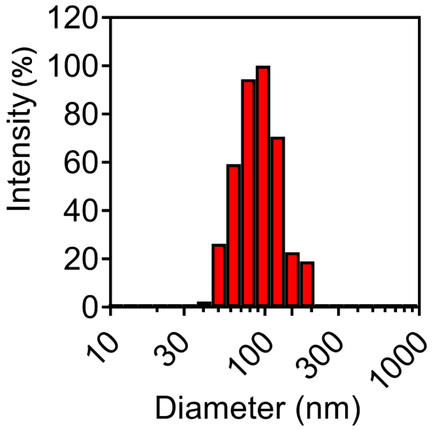

Figure S3. Size distribution of AB@Lip NPs.

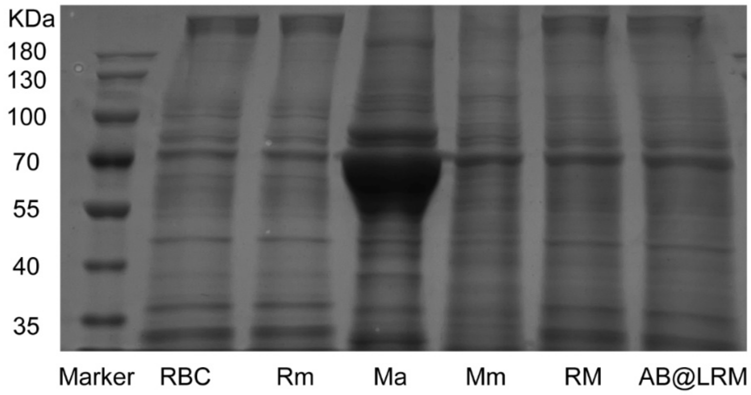

Figure S4. SDS-PAGE protein assay of key proteins in different samples.

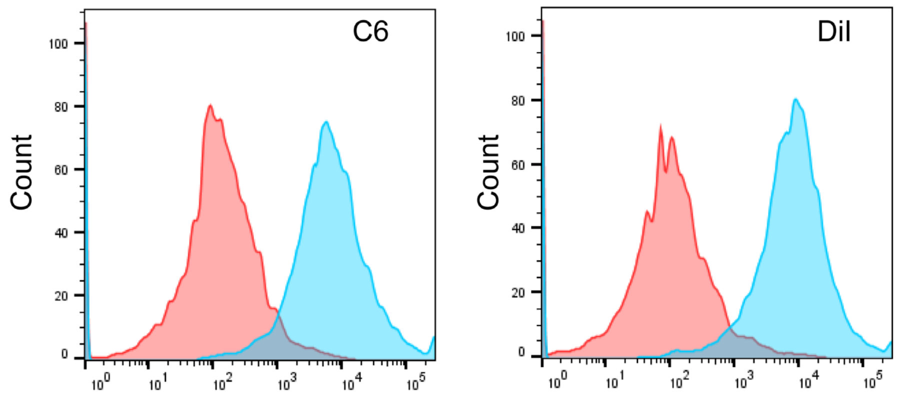

Figure S5. Determination of the composition of AB@LRM by FCM analysis.

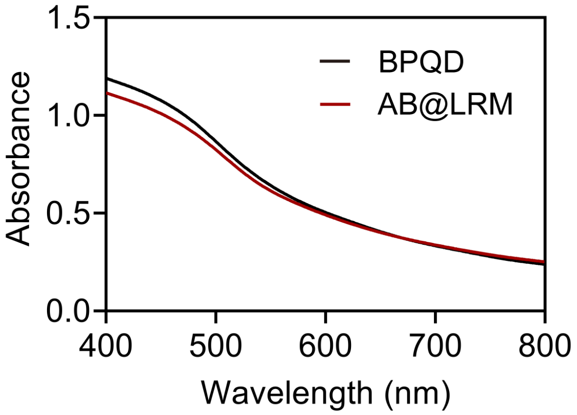

Figure S6. UV-vis spectra of BPQDs and AB@LRM NPs.

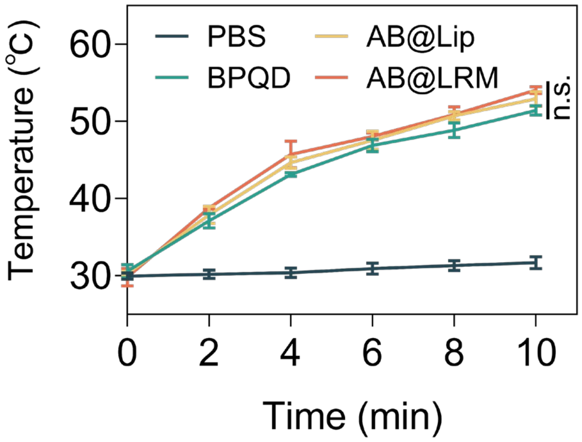

Figure S7. Temperature change of PBS, BPQDs, AB@Lip and AB@LRM with 808 nm laser irradiation (1.5 W/cm^2), respectively (n = 3). BPQD concentration: 20 μg/mL.

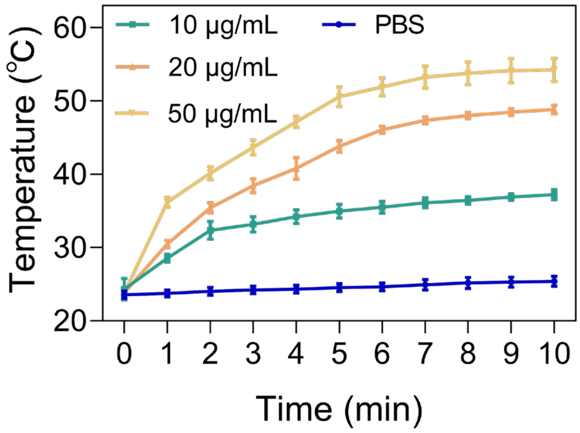

Figure S8. Temperature change curves of AB@LRM (0, 10, 20, and 50 μg/mL) after NIR irradiation (1.5 W/cm^2) (n = 3).

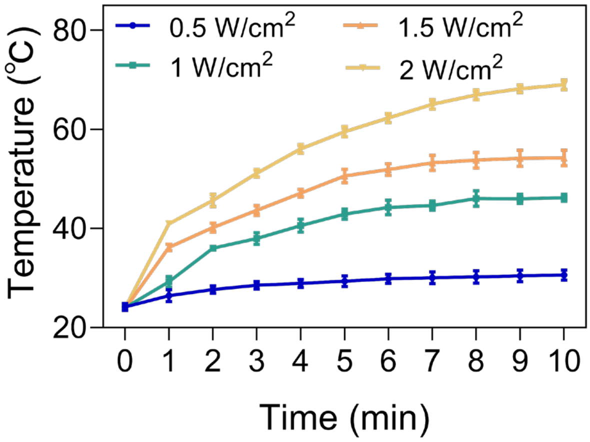

Figure S9. Power density-dependent temperature change curves of AB@LRM (50 μg/mL) after NIR irradiation (0.5, 1.0, 1.5 and 2.0 W/cm^2) (n = 3).

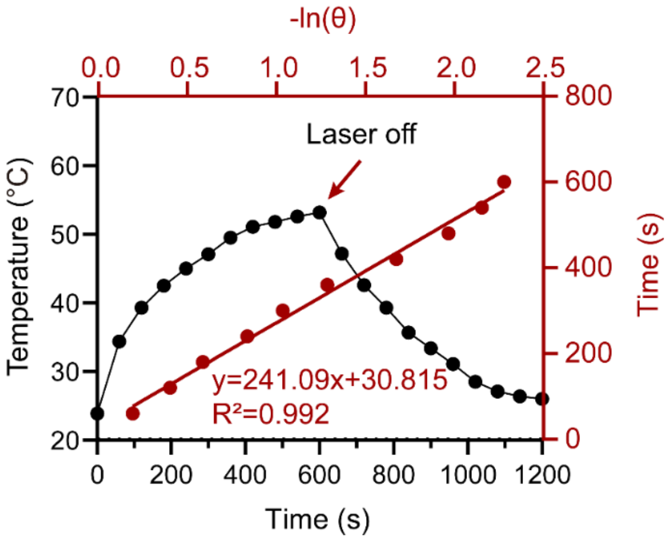

Figure S10. The photothermal curve of AB@LRM (50 μg/mL) during on and off laser (1.5 W/cm^2). Linear cooling time *vs* -ln (θ) of AB@LRM acquired from the photothermal curve.

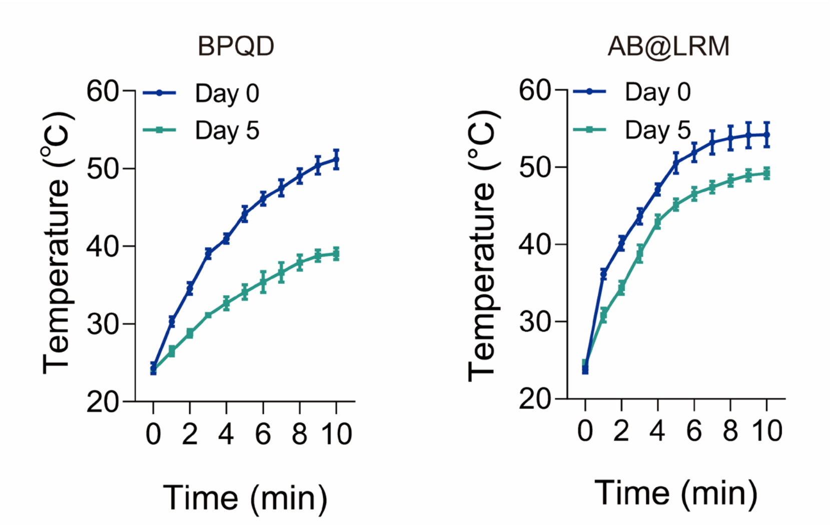

Figure S11. Photothermal curves of BPQDs and AB@LRM in water on day 0 and day 5 (*n* = 3).

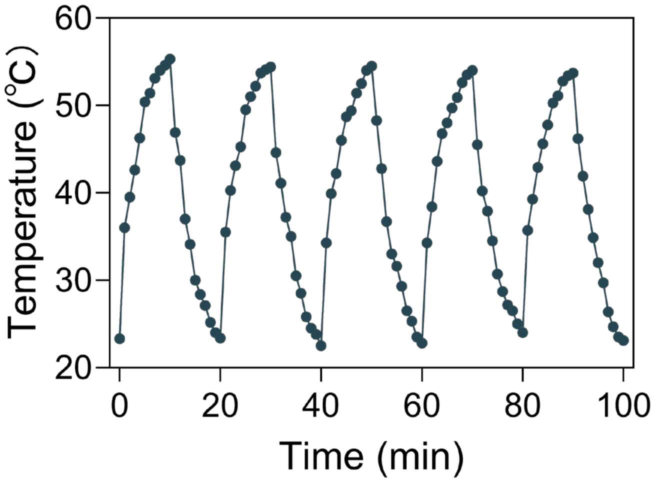

Figure S12. Temperature rise cycle of AB@LRM (50 μg/mL) under NIR irradiation (808 nm, 1.5 W/cm^2, 10 min) followed by natural cooling.

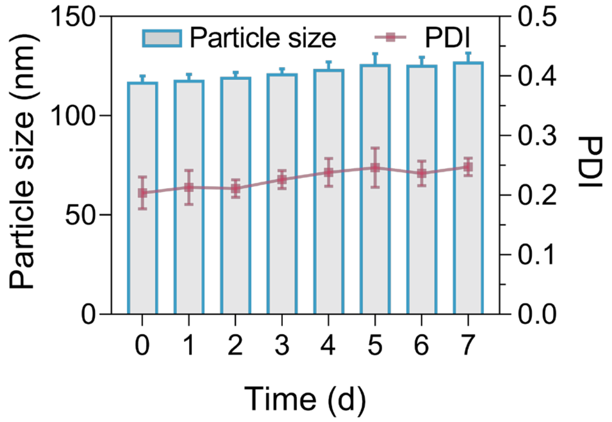

Figure S13. Stability of AB@LRM over time in PBS (*n* = 3).

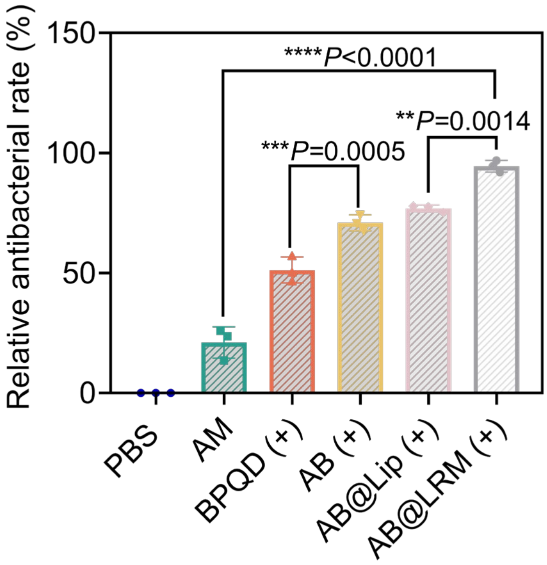

Figure S14. The relative antibacterial rate by plate counting (n = 3). **p < 0.01, ***p < 0.001, ****p < 0.0001.

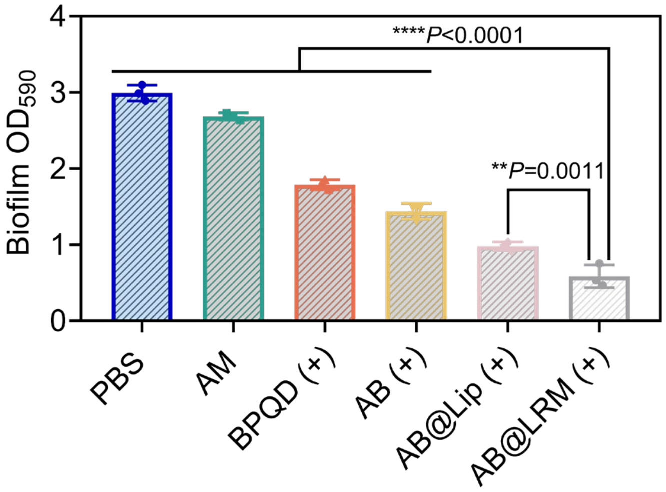

Figure S15. Optical density at 590 nm of biofilms stained with crystal violet (n = 3). **p < 0.01, ****p < 0.0001.

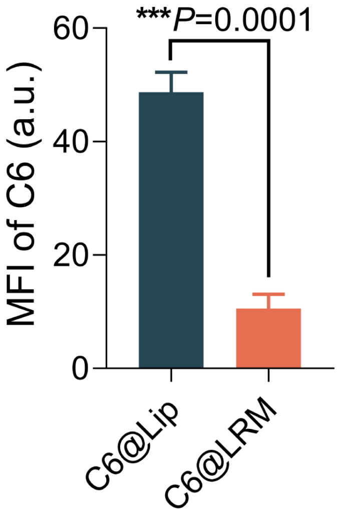

Figure S16. Quantification of the cellular uptake of C6@Lip and C6@LRM in RAW264.7 cells ($n = 3$). ***$p < 0.001$.

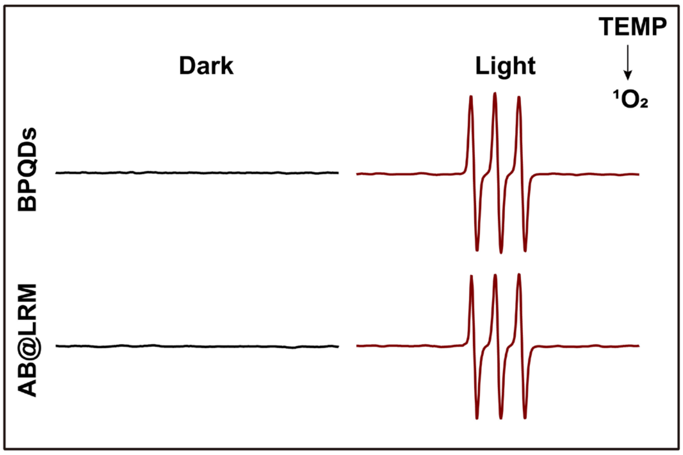

Figure S17. The electron spin resonance (ESR) spectra of BPQD and AB@LRM under NIR laser irradiation (808 nm, 1.5 W/cm^2, 10 min). 2,2,6,6-tetramethylpiperidine (TEMP) was used as the probe.

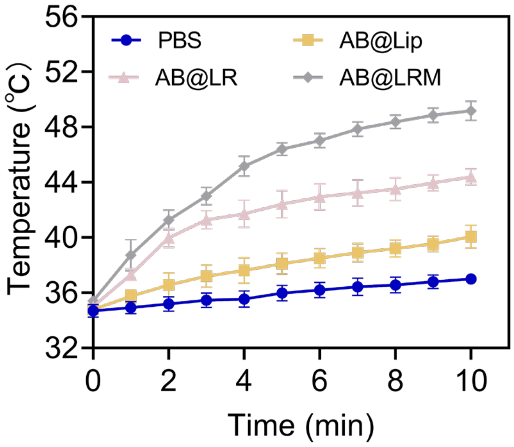

Figure S18. Temperature increase at the infected wound site with 808 nm NIR irradiation (1.5 W/cm^2, 10 min) (n = 5).

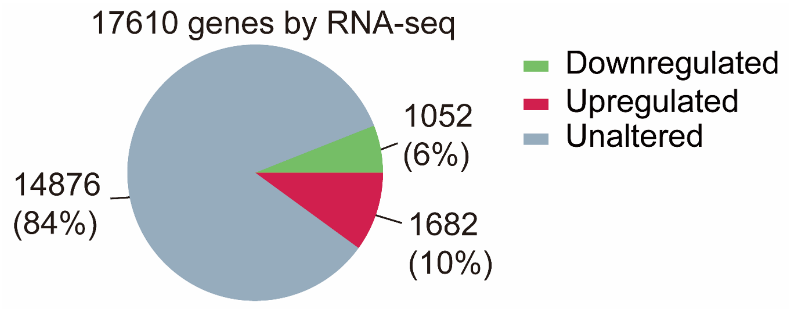

Figure S19. Pie chart revealing the total number of genes with altered expression and the percentage of upregulated/downregulated genes.

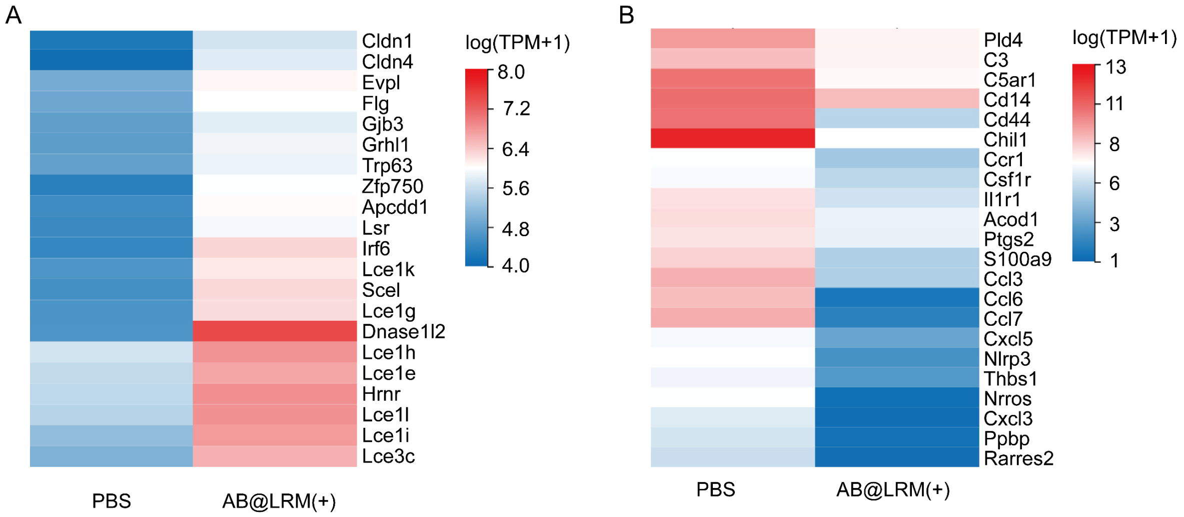

Figure S20. A) Upregulated inflammatory response genes after AB@LRM (+) treatment. B) Downregulated wound healing-related genes after AB@LRM (+) treatment.

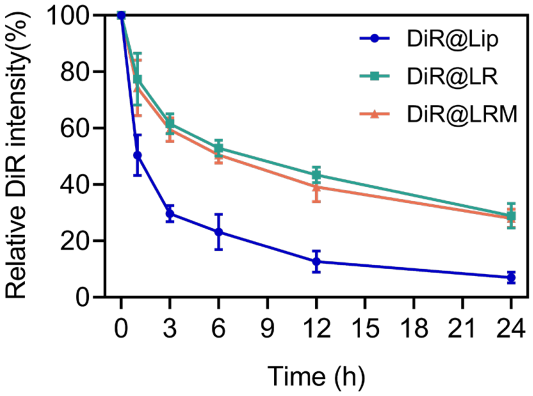

Figure S21. Plasma kinetics of DiR@Lip, DiR@LR, and DiR@LRM in ICR mice after *i.v.* administration ($n = 5$).

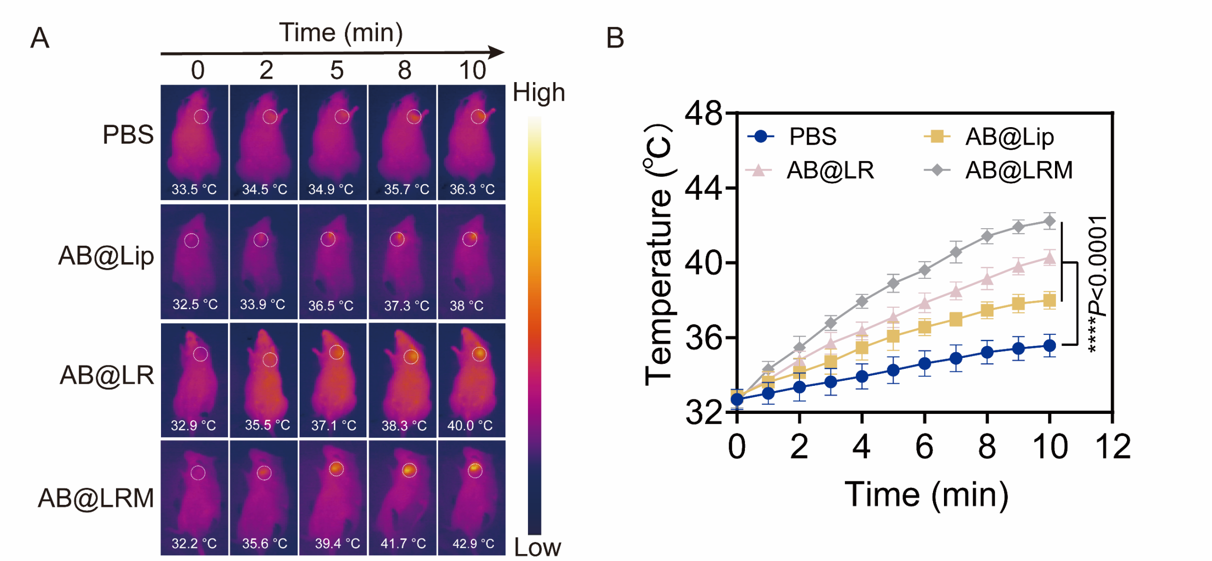

Figure S22. A) Thermal images of the mice suffered from bacterial pneumonia after the injection of PBS, AB@Lip, AB@LR or AB@LRM NPs upon 808 nm laser irradiation. B) Temperature increase at the lung after NIR irradiation (n = 5). ****p < 0.0001.

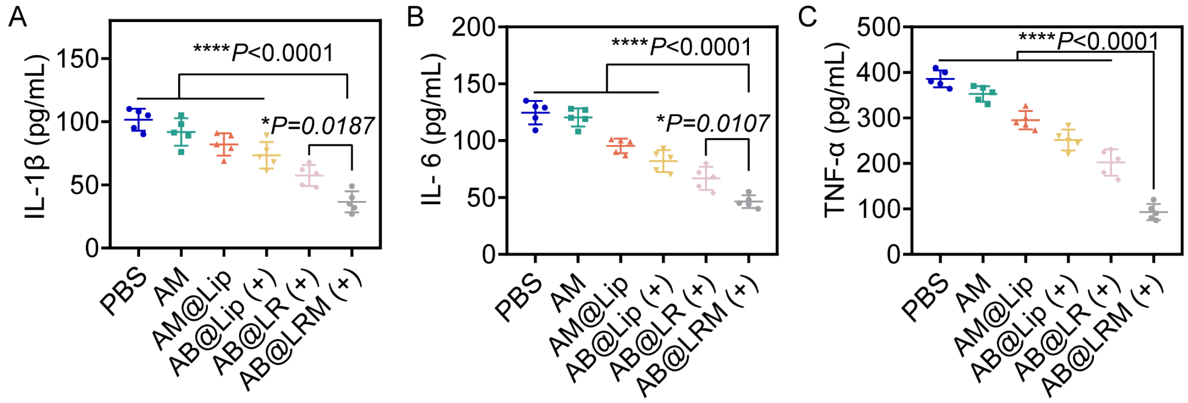

Figure S23. Cytokine levels (IL-1β, IL-6, TNF-α) in serum of mice from various groups (n = 5). *p < 0.05, ****p < 0.0001.

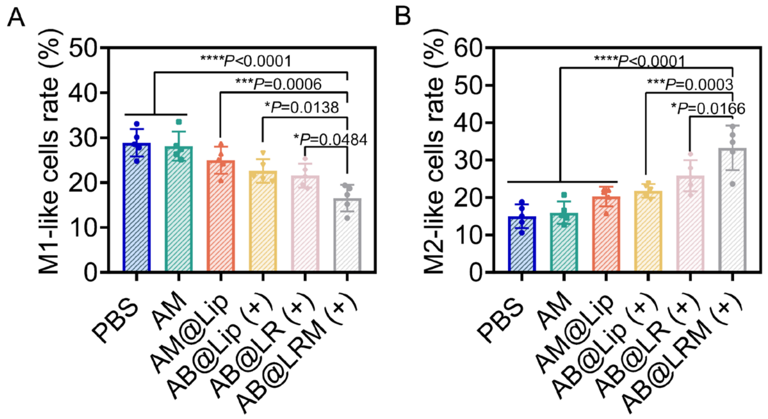

Figure S24. Quantification of M1-like and M2-like macrophage rates after different treatments (n = 5). *$p < 0.05$, ***$p < 0.001$, ****$p < 0.0001$.

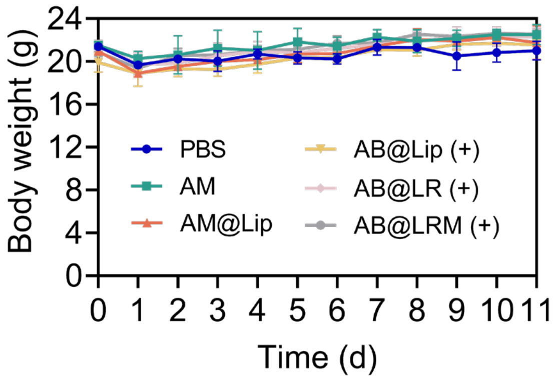

Figure S25. Body-weight curves of the infected wound mice after different treatments (*n* = 5).

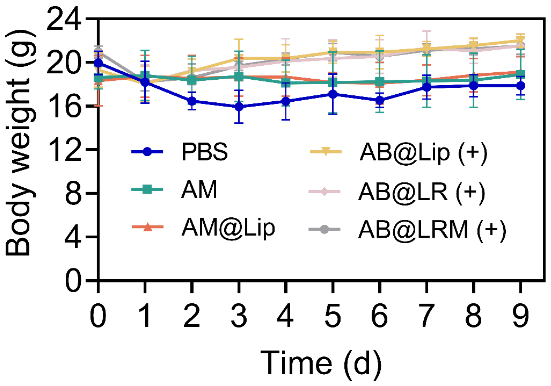

Figure S26. Body-weight curves of mice suffered from bacterial pneumonia after various treatments (n = 5).

References

[1] F. Lu, Z. Li, Y. Kang, Z. Su, R. Yu, S. Zhang, *J Mater Chem B* **2020**, *8*, 10650.
[2] L. Tang, Y. Yin, Y. Cao, C. Fu, H. Liu, J. Feng, W. Wang, X. J. Liang, *Adv. Mater.* **2023**, *35*, e2303835.
[3] L. Ran, B. Lu, H. Qiu, G. Zhou, J. Jiang, E. Hu, F. Dai, G. Lan, *Bioact Materi* **2021**, *6*, 2956.
[4] H. Cao, Y. Gao, H. Jia, L. Zhang, J. Liu, G. Mu, H. Gui, Y. Wang, C. Yang, J. Liu, *Nano Lett.* **2022**, *22*, 7882.
[5] P. You, A. Mayier, H. Zhou, A. Yang, J. Fan, S. Ma, B. Liu, Y. Jiang, *Appl Mater Today* **2022**, *26*, 101386.
[6] G. Gao, Y. W. Jiang, H. R. Jia, F. G. Wu, *Biomaterials* **2019**, *188*, 83.
[7] S. Huang, S. Xu, Y. Hu, X. Zhao, L. Chang, Z. Chen, X. Mei, *Acta Biomater* **2022**, *137*, 199.
